# Improving wheat tolerance to post-flowering heat using matched development stages and field-based reaction norms

**DOI:** 10.1093/jxb/erag188

**Published:** 2026-04-17

**Authors:** Muhammad Yahya, Najeeb Ullah, Jack Christopher, Daniel Cozzolino, Vincent Mellor, Glen Fox, Estelle Kuhn, Karine Chenu

**Affiliations:** Queensland Alliance for Agriculture and Food Innovation (QAAFI), The University of Queensland, Gatton, Queensland 4343, Australia; Queensland Alliance for Agriculture and Food Innovation (QAAFI), The University of Queensland, Brisbane, St Lucia, Queensland 4072, Australia; Agriculture Research Station, Office of VP for Research and Graduate Studies, Qatar University, Doha 2713, Qatar; Queensland Alliance for Agriculture and Food Innovation (QAAFI), The University of Queensland, Gatton, Queensland 4343, Australia; Queensland Alliance for Agriculture and Food Innovation (QAAFI), The University of Queensland, Brisbane, St Lucia, Queensland 4072, Australia; School of Agriculture and Food Sustainability, The University of Queensland, Gatton, Queensland 4343, Australia; Department of Food Science and Technology, University of California Davis, Davis, CA 95616, USA; Université Paris-Saclay, INRAE, MaIAGE, 78350 Jouy-en-Josas, France; Queensland Alliance for Agriculture and Food Innovation (QAAFI), The University of Queensland, Gatton, Queensland 4343, Australia; ARC Training Centre in Predictive Breeding, The University of Queensland, St Lucia, Queensland 4072, Australia; ARC Hub for Engineering Plants to Replace Fossil Carbon, The University of Queensland, St Lucia, Queensland 4072, Australia; University College Dublin, Ireland

**Keywords:** Cereal, climate change, crop adaptation, genotypic variation, global warming, grain filling, heat stress, thousand grain weight

## Abstract

Post-flowering heatwaves impact individual grain weight (IGW) and grain quality of wheat (*Triticum aestivum*). Here, the effect of post-flowering heatwaves on IGW, yield, and grain protein content (GPC) were examined in 25 wheat genotypes grown in irrigated field trials across locations, seasons and sowing dates. The photoperiod-extension method (PEM) was employed with measurements on individual spikes to synchronize flowering among genotypes, ensuring heat impacts were assessed at similar developmental stages. Adjacent to most PEM trials, genotypes were cultivated in machine-harvested field plots to evaluate crop performance using a more conventional screening method. Heat stress significantly reduced IGW and increased GPC in PEM trials, with impacts not as clearly discernible in conventional plots. Across genotypes and traits, heat responses (slope of the reaction norms) were negatively correlated with trait values observed in non-stressed conditions (intercept). The level of heat stress each genotype could endure before reaching critical IGW (30 mg), yield (250 g m^−2^), or GPC (15%) thresholds revealed high genotypic variability and highly tolerant genotypes. The findings provide insights for improving heat responses in crop models and enhancing genotype selection for climate-resilient breeding.

## Teaser

Reaction norms to post-flowering heat estimated with the photoperiod-extension method effectively revealed variation in heat tolerance across wheat genotypes that could be useful to develop more tolerant cultivars.

## Introduction

Wheat is a primary carbohydrate source in human diets worldwide (De Punder and Pruimboom, 2013; Khalid *et al.*, 2023). However, in recent decades, wheat-growing regions, such as Australia, have been experiencing an increasing frequency of heat-stress events, particularly during the grain-filling phase (Ababaei and Chenu, 2020). With global warming, the frequency of heatwaves is projected to further increase in future decades, with major impacts on wheat crops (Lobell *et al.*, 2015; Collins and Chenu, 2021; Fernie *et al.*, 2022; He *et al.*, 2022).

High temperatures impact different physiological processes, depending on the timing and duration of their occurrence (Ravindra *et al.*, 2025). Higher average temperatures accelerate crop development, shorten the crop cycle, and reduce both biomass and yield potential (e.g. Zheng *et al.*, 2012; Ruiz-Vera *et al.*, 2015). Warmer temperatures also increase plant evaporation requirement, thus reducing transpiration efficiency (e.g. Kemanian *et al.*, 2005; Lobell *et al.*, 2013; Chenu *et al.*, 2018; Fletcher *et al.*, 2018; Collins *et al*., 2021). By contrast, heatwaves (i.e. supra-optimal temperatures) have a direct stressful impact on wheat productivity. Heat stress occurring before flowering affects pollen viability, impairs fertilization, and limits grain set (Wollenweber *et al.*, 2003; Barnabás *et al.*, 2008; Sharma *et al.*, 2008). Heat stress during grain filling induces premature leaf senescence, limiting photosynthetic capacity of plants (Lopes *et al.*, 2012; Pinto *et al.*, 2016) and disrupting the translocation of assimilate to developing grains (Talukder *et al.*, 2014), which ultimately results in reduced grain size and overall grain yield (Chenu and Oudin, 2019; Hütsch *et al.*, 2019; Girousse *et al.*, 2021; Yahya *et al.*, 2022; Ullah *et al.*, 2023, 2024).

While grain yield remains a primary determinant of farm profitability, grain quality traits such as individual grain weight (IGW), grain protein content (GPC), and grain protein composition are also important, particularly for milling and bread-making quality (Abdipour *et al.*, 2016). Post-flowering heat has been found to reduce grain size with associated increase in GPC (Labuschagne *et al.*, 2009; Balla *et al.*, 2011; Liu *et al.*, 2016). Post-flowering heat stress also alters grain protein composition, typically reducing the glutenin-to-gliadin ratio (Branlard *et al.*, 2015; Narwal, 2022), which ultimately adversely affects flour quality and dough properties (Bencze *et al.*, 2004; Tomás *et al.*, 2020; Narwal, 2022).

Previous studies have employed a range of methods to evaluate genotypic responses to heat stress. For example, late sowing dates have been adopted to push the flowering and grain filling stages into later periods when damaging heat events are more likely. Field heat chambers such as portable polyethylene tents have been deployed to induce heat stress (Thistlethwaite *et al.*, 2020; Telfer *et al.*, 2021). As different genotypes develop at varying rates, their growth stages typically differ when heatwaves occur. However, the developmental stage at which heat stress occurs can greatly affect the type and severity of damage that occurs (Stone and Nicolas, 1995; Chenu and Oudin, 2019; Mirosavljević *et al.*, 2024). Controlled environment experiments can ensure uniform exposure of genotypes to heat stress at targeted developmental stages. However, results from these experiments may not extrapolate to the field in part due to differences in plant density, border effects, or differences in soil temperature around the root system (Poiré *et al.*, 2010; Rebetzke *et al.*, 2014). Targeting similar development stages is also possible in the field with careful timing of placement of heat chambers or tents, but these may impact other microclimatic factors, such as radiation, wind, humidity, and CO_2_ levels (Telfer *et al.*, 2018; Thistlethwaite *et al.*, 2020).

To address these challenges, the photoperiod-extension method (PEM) was developed to assess the genotypic responses to natural field heat stress events at similar developmental stages (Ullah *et al.*, 2023). The PEM approach extends the photoperiod using artificial lights, resulting in a developmental gradient in each row of plants, with those closer to light flowering earlier than those farther away (Frederiks *et al.*, 2012; Ullah *et al.*, 2023). This method enables a focus on plants flowering at the same time, irrespective of genotype maturity type, allowing the evaluation of genotypes for heat tolerance under natural field conditions at matched developmental stages.

The aim of the current study was to evaluate the response of 25 diverse genotypes to post-flowering heatwaves. Genotypes included widely cultivated high-performing spring Australian cultivars, lines described as heat tolerant germplasm from the International Center for Maize and Wheat Improvement (CIMMYT), and donor parents from a multi-reference nested association mapping (MR-NAM) population (Richard, 2017). These genotypes were grown with the PEM and in adjacent conventional plots at different sowing dates in irrigated field. The plasticity of each genotype to heatwaves was investigated in terms of reaction norms (linear regressions) for yield, IGW, and GPC in response to the number of post-flowering hot days (*T*>32 °C, between 0 °Cd and 500 °Cd after flowering). Genotypic levels of heat tolerance were further characterized by estimating the number of hot days the genotype can endure before its grain yield dropped below the benchmark of 250 g m^−2^, its average IGW dropped below 30 mg, and its GPC increased above 15%. The results from the PEM trials, where measurements were done on single stem (i.e. organ level), and from conventional plot trials, where measurements were conducted at the crop level (i.e. machine harvest of the plot), were compared.

## Materials and methods

### Growing conditions and experimental designs

Field trials were conducted at Gatton (27°34′50″S, 152°19′28″E), Warwick (28°12′40″S, 152°06′06″E), and Tosari (27°49′09″S, 151°26′15″E) between 2018 and 2020 (Table 1). Two sowing times were used: one with a standard sowing date (‘s1’) when post-flowering heat damage was less likely and another sown later (‘s2’) to maximize the chance of heat stress after flowering. Trials were named after the location, year, and sowing (e.g. ‘GAT18s1’). Where possible, genotypes were cultivated in both PEM trials (eight trials) and conventional yield plots (nine trials) adjacent to each other with the same management practices. However, in 2018, only one conventional field trial was conducted (GAT18s2) for four PEM trials (GAT18s1, GAT18s2, WAR18s1, WAR18s2), while by contrast, in 2020, only conventional trials were established (i.e. GAT20s1, GAT20s2, WAR20s1, WARs2; Table 1).

**Table 1.** Trial characteristics

Trial	Location	Method	Sowing	Irrigation	Mean *T* (°C)	Max *T* (°C)	VPD (kPa)	Radn (MJ m^−2^)	DTF (d)	Yield (g m^−2^)	Grain number (m^−2^)					
GAT18s1	Gatton	PEM (Spike)	3/07/2018	Full	19.3	26.3	0.74	23.8	73	NA	NA	45.7±0.4	4.9±0.1	10.8±0.1	0	HET1
GAT18s2	Gatton	PEM (Spike)	31/08/2018	Full	23.1	31.4	1.38	23.2	53	NA	NA	34.2±0.4	3.8±0.1	11.1±0.9	8	HET2
		Plot (Crop)							59±0.2	216±5	6403±144	33.7±0.3	4.0±0.05	11.8±0.1		
WAR18s1	Warwick	PEM (Spike)	16/07/2018	Full	18.6	25.6	0.84	17.7	73	NA	NA	46.2±0.3	5.6±0.1	12.4±0.1	0	HET1
WAR18s2	Warwick	PEM (Spike)	12/09/2018	Full	22.0	30.3	1.52	21.2	52	NA	NA	27.9±0.4	3.9±0.1	14.2±0.1	5	HET2
GAT19s1	Gatton	PEM (Spike)	9/07/2019	Full	19.5	28.7	1.40	16.8	71	NA	NA	38.3±0.2	4.5±0.1	11.7±0.1	4	HET2
		Plot (Crop)							83±0.2	188±3	5049±105	36.7±0.2	5.7±0.0	15.4±0.1		
GAT19s2	Gatton	PEM (Spike)	3/09/2019	Full	23.7	33.8	2.23	20.8	59	NA	NA	13.7±0.4	2.5±0.1	18.0±0.1	14	HET3
		Plot (Crop)							61±0.2	93±2	3844±113	24.9±0.2	4.2±0.0	16.8±0.1		
TOS19s1	Tosari	PEM (Spike)	16/07/2019	Partial	21.1	30.2	1.26	17.7	79	NA	NA	26.5±0.2	4.1±0.0	15.6±0.1	8	HET2
		Plot (Crop)							87±0.2	269±2	9929±131	27.4±0.2	4.8±0.0	17.6±0.1		
TOS19s2	Tosari	PEM (Spike)	6/09/2019	Partial	26.5	36.7	2.27	22.2	62	NA	NA	18.4±0.2	3.2±0.0	17.2±0.1	21	HET3
		Plot (Crop)							65±0.3	62±1	2545±59	24.2±0.1	4.4±0.1	18.4±0.1		
GAT20s1	Gatton	Plot (Crop)	26/05/2020	Full	17.9	26.8	1.08	17.3	89±0.2	256±3	9470±293	27.6±0.3	4.4±0.1	16.0±0.1	0	HET1
GAT20s2	Gatton	Plot (Crop)	4/08/2020	Full	22.2	31.2	1.35	19.7	69±0.2	168±3	4656±154	35.6±0.3	5.3±0.1	15.2±0.1	7	HET2
WAR20s1	Warwick	Plot (Crop)	8/06/2020	Full	19.3	26.3	0.74	13.6	108±0.2	476±3	12 917±172	37.4±0.3	4.9±0.0	13.2±0.1	0	HET1
WAR20s2	Warwick	Plot (Crop)	12/08/2020	Full	23.1	31.4	1.38	11.6	73±0.3	369±4	10 848±136	34.0±0.3	4.3±0.0	12.6±0.1	1	HET2

Trial characteristics included location, method used (PEM or conventional plot), harvest (spike or crop level), date of sowing, irrigation treatment, mean air temperature, maximum daily temperature, mean daytime vapor pressure deficit (VPD), mean daily radiation (Radn), as well as the trial mean and standard error for days from sowing to flowering (DTF), yield, grain number (per m^−2^), grain protein amount per grain, grain protein content (percentage by weight), number of post-flowering hot days (*T*>32 °C) between 0 °Cd and 500 °Cd after flowering for PEM trials, and heat environment type (HET). Environmental means were calculated from sowing to harvest. PEM, photoperiod-extension method.

The PEM used LED lamps (CLA LT401, 9 W T40 LED lamp, 3000 K 760 lm) with a lumen efficiency of ≥80 at the end of each row or plot. The lights were placed around 1 m above-ground level and 0.8 m apart from each other. These lamps extended the light period to 20 h. The intensity of light decreases with the square of the distance between the lights along the test row. For example, at 1 and 2 m distances from the light source, quantum flux density can fall by 10 and 45 times, respectively, with little or no impact after 3 m (Niinemets and Keenan, 2012). The plants nearest to the light received the most supplemental light, while plants at the opposite end of the row had little or no light impact. The decreasing light effect resulted in a developmental gradient along each row of plants, with plants close to the light flowering earlier than those farther away. This made it possible to select plants of differing genotype that flowered on the same day (synchronized flowering) irrespective of the genotype maturity type.

For both the PEM and conventional plot trials, each trial was organized using a randomized complete block design with four replicates. All trials, except those at Tosari, received full irrigation. Plants were irrigated with (i) a boom irrigator in plot trials at Gatton and Tosari (2018–2020), as well as in PEM trials at Tosari (2019), (ii) wobbler sprinklers in PEM trials at Gatton (2018–2020) and Warwick (2018), (iii) drip irrigation in PEM and plot trials at Warwick (2020). Because of water-supply constraints, irrigation in Tosari trials was restricted to the pre-flowering period, with no further irrigation applied after flowering. Plants were cultivated under non-limiting nutrient levels, with 120 kg ha^−1^ of urea applied prior to sowing and 40 kg ha^−1^ of Starter Z® containing 10.5% N, 19.5% P, 2.2% S, and 2.2% Zn at sowing time. Standard agronomic practices, including weeding, disease management, and pest management, were applied in all trials.

In PEM trials, wheat genotypes were manually planted in a single 5 m row with 33 cm row spacing in 2018 and 2019. In plot trials, genotypes were machine planted and cultivated in 2×6 m plots at Gatton and Warwick in 2018 and 2020, and in 1×6 m plots at Tosari and Gatton in 2019. All plot trials were planted with a row spacing of 25 cm, a target plant population density of 130 plants per square metre and four replications for each genotype. In Gatton (2018), due to unavailability of irrigation water supply, only late sowing plots (s2) were established.

### Genotypes

Twenty-five wheat (*Triticum aestivum* L.) genotypes (Supplementary Table S1) with varying phenology and heat adaption were grown in all the conventional-plot and PEM trials. These included (i) Australian spring-type cultivars Suntop, Spitfire, Gregory, Janz, Hartog, EGA Wylie, Corack, Yitpi, Mace, and Scout, (ii) genotypes from CIMMYT described as heat tolerant, as well as (iii) donors of a multi-reference parent nested association mapping (MR-NAM) population (Richard, 2017; Christopher *et al.*, 2021), which was developed for screening heat and drought tolerance.

### Plant measurements

For each PEM trial approximately 20 stems per replicate per genotype were tagged at flowering (Zadoks growth stage 65; Zadoks *et al.*, 1974). At maturity, spikes from tagged stems were manually harvested (‘single spike’ harvest) and processed to assess IGW and GPC. In each plot trial, plots were machine harvested using a specialized small plot harvester at maturity (whole-plot harvest) when the grain moisture content was approximately 11%. Grain number and yield for each individual plot were estimated as grains per square metre (grains m^−2^) and grams per square metre (g m^−2^), respectively. IGW of each plot was estimated from two or three subsamples of 100 grains.

### Estimation of grain protein content and amount

In all PEM and plot trials and for all replicates, grain samples of approximately 5 g each were scanned using a Fourier transform near-infrared spectroscopy (FT-NIR) (Chen *et al.*, 2021) instrument (Bruker, Germany). The FT-NIR spectra were recorded over the range of 3952–11 520 cm^−1^, with a resolution of 4 cm^−1^. To calibrate a model estimating grain protein content based on the FT-NIR spectrum, a subset of 230 representative samples was selected using the Kennard–Stone algorithm (Ferreira *et al.*, 2021). The GPC of the selected 230 samples was determined using the combustion method in a LECO analyser (USA) (Carter *et al.*, 2012), where the total nitrogen content (N) was measured and converted into GPC using the multiplication factor of 5.7 (GPC=N×5.7; Rossmann *et al.*, 2019; Fan *et al.*, 2025). Partial least squares (PLS) regression was used to develop the calibration model.

Grain protein amount (GP; mg grain^−1^) was estimated by multiplying the IGW (mg) with GPC (%) and dividing it by 100.

### Weather data

Each year, a weather station (Campbell Scientific) was installed at each site to measure weather data every minute, and record averages every 10 min. Incident light was measured using an Apogee SP-110 pyranometer for solar radiation, and an Apogee SQ-110 sensor for photosynthetically active radiation, both positioned at a height of 1.5 m above the ground. Air temperature and relative humidity were measured with HMP60 probes (Vaisala INTERCAP®), also placed 1.5 m above the ground.

For each replicate, the number of warm/hot days were calculated for (i) days with a maximum temperature >28 °C occurring between 300 °Cd and 200 °Cd before flowering, and for (ii) days with a maximum temperature >32 °C occurring between 0 °Cd and 500 °Cd after flowering, as suggested by Ullah *et al.* (2024).

The environmental characteristics of each trial are presented in Table 1.

### Statistical analyses

Data in each PEM and plot trial were corrected for spatial heterogeneity using the spatial analysis of field trials with splines (SpATS) method (Rodríguez-Álvarez *et al.*, 2018). This approach accounts for within-trial environmental variability by fitting a two-dimensional spatial trend across rows and columns using penalized splines with a separation of anisotropic penalties (SAP) algorithm. SpATS was applied on the row–column design, but the analysis on the corrected data used a randomized complete block design. The spatial model was as follows:


(1) $$y_{ij}=f(\mathrm{ro}w_{i},\;\;\mathrm{co}l_{i})+\mu +\varepsilon_{ij}$$

where row*_i_* and col*ᵢ* stand for the plot row and column number in the trial layout, *f*(row*_i_*, col*_i_*) is a function representing a smooth bivariate surface, μ is overall mean and ɛ*_ij_* is the residual error.

Following the spatial correction, linear models were applied to study (i) the response of traits (i.e. grain yield, IGW, GPC) to the number of days with a maximum temperature >32 °C between 0 °Cd and 500 °Cd after flowering, and (ii) the relationship between GPC and either grain yield or IGW. As linear regressions were significantly different depending on whether trials were conducted with the PEM or plot method, these analyses were conducted separately. Linear models were fitted for each genotype, as follows:


(2) $$y=\beta_{0}+\beta_{1}\times x+\varepsilon$$

where *y* is the spatially corrected value of the studied trait (i.e. grain yield, IGW, or GPC); *x* is the variable (i.e. number of hot days (*T*>32 °C) between 0 °Cd and 500 °Cd after flowering, grain yield or IGW); β_0_ is the intercept; β_1_ is the slope; and ε is the residual error.

To characterize the heat tolerance of each genotype, the number of hot days the genotype can endure before (i) dropping below 250 g m^−2^ for grain yield (a threshold chosen for its economic relevance in Australia), (ii) dropping below 30 mg for IGW, and (iii) GPC increased to 15%, was calculated from the linear regressions (Eq. 2) for both the PEM and plot methods separately.

Full-factorial analysis of variance was performed on spatially corrected data to evaluate the trial, sowing (s1 or s2), and genotype effects as well as their interactions for each studied trait (i.e. yield, grain number, IGW, grain protein (protein mg grain^−1^), and GPC. The factors Location–Year, Sowing, and Genotype were considered as fixed effects, and all two-way and three-way interactions were included in the analysis.

All the statistical analyses and graphs were performed with R version 4.2.0 (R Core Team, 2018).

## Results

### A wide range of post-flowering heat environments

A total of 12 environments (10 fully irrigated and two partially irrigated) were studied across three years, three sites, and ‘standard’ (s1) or late sowing dates (s2). Trials were conducted with (i) the PEM to study spikes with synchronized phenology and/or (ii) conventional field plots (i.e. studied at the crop level without synchronized phenology). Each trial received varying levels of heat, with most heatwaves occurring post flowering (Table 1). Across all trials, mean daily maximum temperatures (*T*_max_) ranged from 25.6 °C to 36.7 °C, mean daily temperatures from 17.9 °C to 26.5 °C and mean daytime vapor pressure deficit (VPD) varied from 0.74 kPa to 2.27 kPa.

As intended, late-sown crops (s2) were typically subjected to more hot days than standard-sown crops (s1). They experienced higher averaged daily maximum (*T*_max_) and mean (*T*_mean_) temperature, as well as higher daytime evaporative demand (VPD) and radiation levels (Table 1). Sowing time had a major and statistically significant impact on yield and its components (Supplementary Table S2). Crops cultivated in trials with warmer temperature typically developed faster and yielded less. For instance, late-sown crops had a pre-flowering phase 12 to 21 d shorter than standard-sown crops and yielded between 62 g m^−2^ and 369 g m^−2^ less than standard-sown crops, at a given site in a given year (Table 1).

Trials were classified into heat environment types (HETs) based on the number of post-flowering hot days (*T*>32 °C) that the genotypes experienced between 0 °Cd and 500 °Cd after flowering (Table 1). HET1 environments experienced no post-flowering hot days, typically having cooler pre- and post-flowering resulting in slower phenological development, for example with days to flower ranging from 73 d to 79 d. HET2 environments experienced moderate post-flowering heat stress (1–9 hot days) and had flowering 8–21 d earlier (in days after sowing) than HET1. HET3 environments faced severe post-flowering heat stress (≥10 hot days). Compared with ‘non-stressed’ HET1 trials (i.e. WAR20s1, GAT20s1), HET3 trials (i.e. TOS19s2, GAT19s2) experienced 21.5% higher mean temperatures, 18.9% higher *T*_max_, and 67.2% greater VPD.

### Post-flowering heatwaves reduced individual grain weight and yield, while increasing grain protein content

**Fig. 1.**
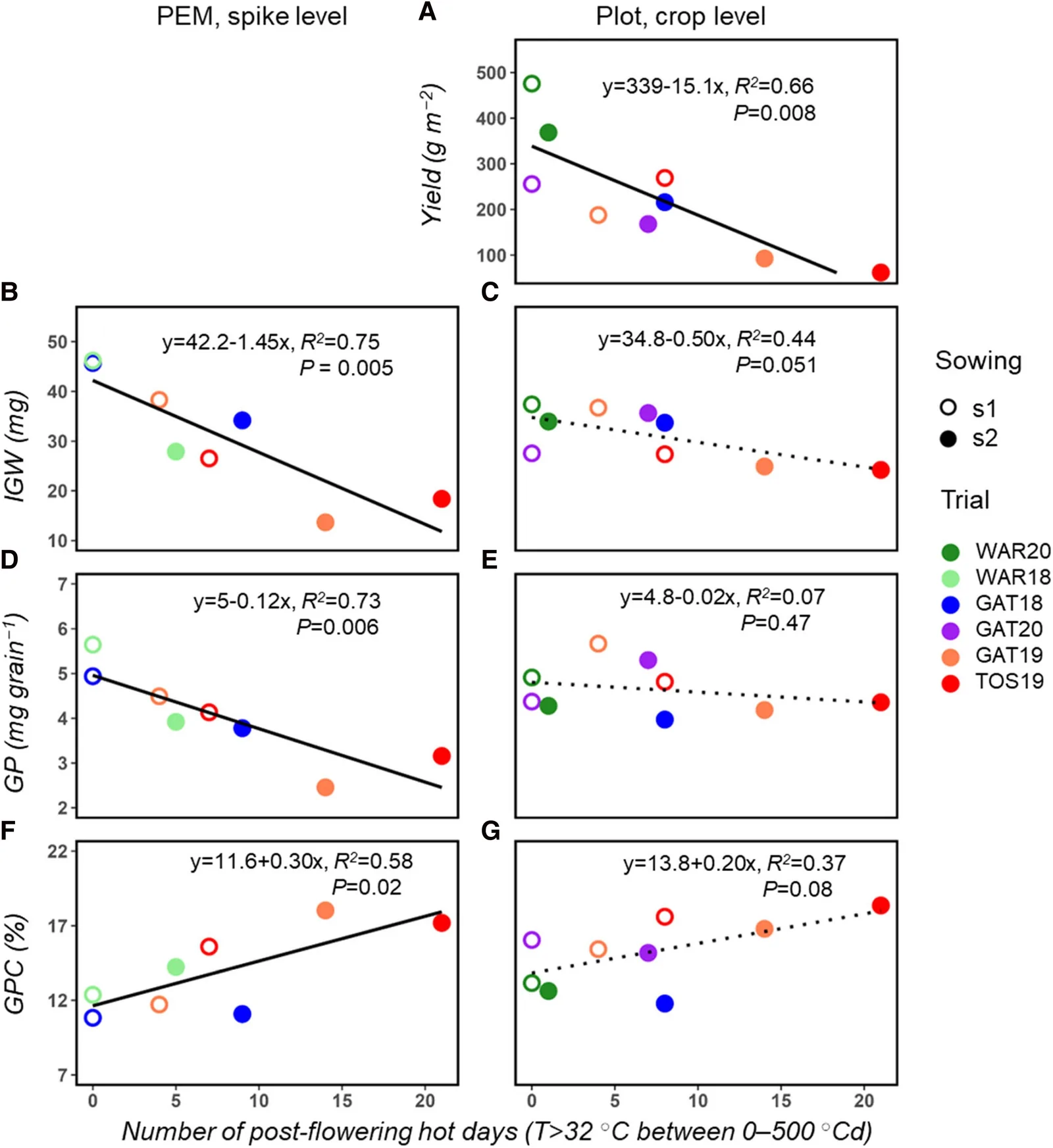
Impact of post-flowering hot days (*T*>32 °C, between 0 °Cd and 500 °Cd after flowering) on (A) grain yield (g m^−2^), (B, C) individual grain weight (IGW, mg), (D, E) grain protein amount (GP, mg grain^−1^), and (F, G) grain protein content (GPC, %), in photoperiod-extension method (PEM) trials (spike level; B, D, F) and plot trials (crop level; A, C, E, G). Each data point represents the trial mean value, i.e. average for the 25 genotypes. Location×year combinations are distinguished by colour: WAR20 (forest green), WAR18 (light green), GAT18 (blue), GAT20 (purple), GAT19 (coral), and TOS19 (red). Sowing times are indicated by symbols, open circles for conventional sowing (s1) and filled circles for late sowing (s2). Linear relationships with a *P*-value≤0.05 are represented by solid lines, while others are presented with dotted lines.

Post-flowering heatwaves impacted genotypes both in (i) the PEM trials, which focused on individual spikes (i.e. organ level) with synchronized phenology irrespective of genotype maturity types, and (ii) conventional plots, which focused on the crop level, with varying phenology across genotypes and tiller ranks. The number of post-flowering hot (*T*>32 °C) days was negatively associated with grain yield, IGW, and grain protein amount per grain (GP), while being associated with greater GPC values (Fig. 1). The effects were significant for all these studied traits in the PEM, while they were only significant for grain yield in the plot trials. The focus on different levels (organ vs crop level) and on heatwaves impacting genotypes at a synchronized phenology (PEM) or not (conventional plots) also resulted in contrasting ranges of phenotypic variations, with wider ranges observed in the PEM (i.e. synchronized spikes) than in plots (i.e. unsynchronized crops). For example, IGW ranged from 13.0 mg to 46.7 mg in the PEM compared with 24.2–27.7 mg in plots (Fig. 1B, C), and GPC ranged between 10.8% and 17.9% in the PEM compared with 11.8–18.3% in plots (Fig. 1F, G). Some of these differences could also be due to the fact that more of the smaller grains (10–20 mg), which are typically those with higher GPC, are often lost when harvesting plots with mechanical harvesters but retained when threshing spike samples from the PEM.

In plot trials, trial mean grain yield (i.e. average for the 25 genotypes) was negatively correlated with heat stress and decreased by 15.1 g m^−2^ for each additional post-flowering hot day (*T*>32 °C) occurring between 0 °Cd and 500 °Cd after flowering (*P*<0.01, *R*^2^=0.66; Fig. 1A). In HET3 trials with severe post-flowering heatwaves, yield was reduced by an average 79% compared with HET1 trials with no heatwave (Fig. 1A).

A strong impact from the number of post-flowering hot days (*T*>32 °C) was observed on trial-mean IGW of single spikes from PEM trials (*P*<0.01, *R*^2^=0.75; Fig. 1B), while the impact was not significant at the crop level in plot trials (*P*=0.051, *R*^2^=0.44; Fig. 1C). In the PEM, IGW declined by 1.5 mg for every extra hot day after flowering, while it tended to decline by 0.5 mg per hot days in plot trials (Fig. 1B). Concordantly, IGW between HET3 and HET1 environments was reduced on average by 64% in PEM trials but only 24% in plot trials.

Frequent occurrence of post-flowering hot days resulted in significant reduction in protein amount per grain (GP; *P*<0.01, *R*^2^=0.73; Fig. 1D) at a rate of 0.12 mg grain^−1^ per additional hot day in PEM trials (Fig. 1). Although GP was reduced by post-flowering heatwaves, the relative GPC (expressed as a percentage of protein mass to grain mass) increased significantly (*P*<0.05, *R*^2^=0.58; Fig. 1F) at a rate of 0.30% GPC for each extra post-flowering hot day in PEM trials. GPC increased from 11.6% (HET1 trials) to 18.0% in GAT19s2 trial in HET3 (14 hot post-flowering days), reflecting a 6.4% increase in GPC (Fig. 1F). In comparison with PEM results, in plot trials, trial-mean GP tended to decrease at a slower rate of 0.02 mg grain^−1^ per post-flowering hot day (*P*=0.47, *R*^2^=0.07), and GPC also tended to increase at a slower rate of 0.19% per post-flowering hot day (*P*=0.08, *R*^2^=0.37) (Fig. 1E, G).

### A large range of genotypic variation in heat tolerance for individual grain weight, yield and grain protein content

**Fig. 2.**
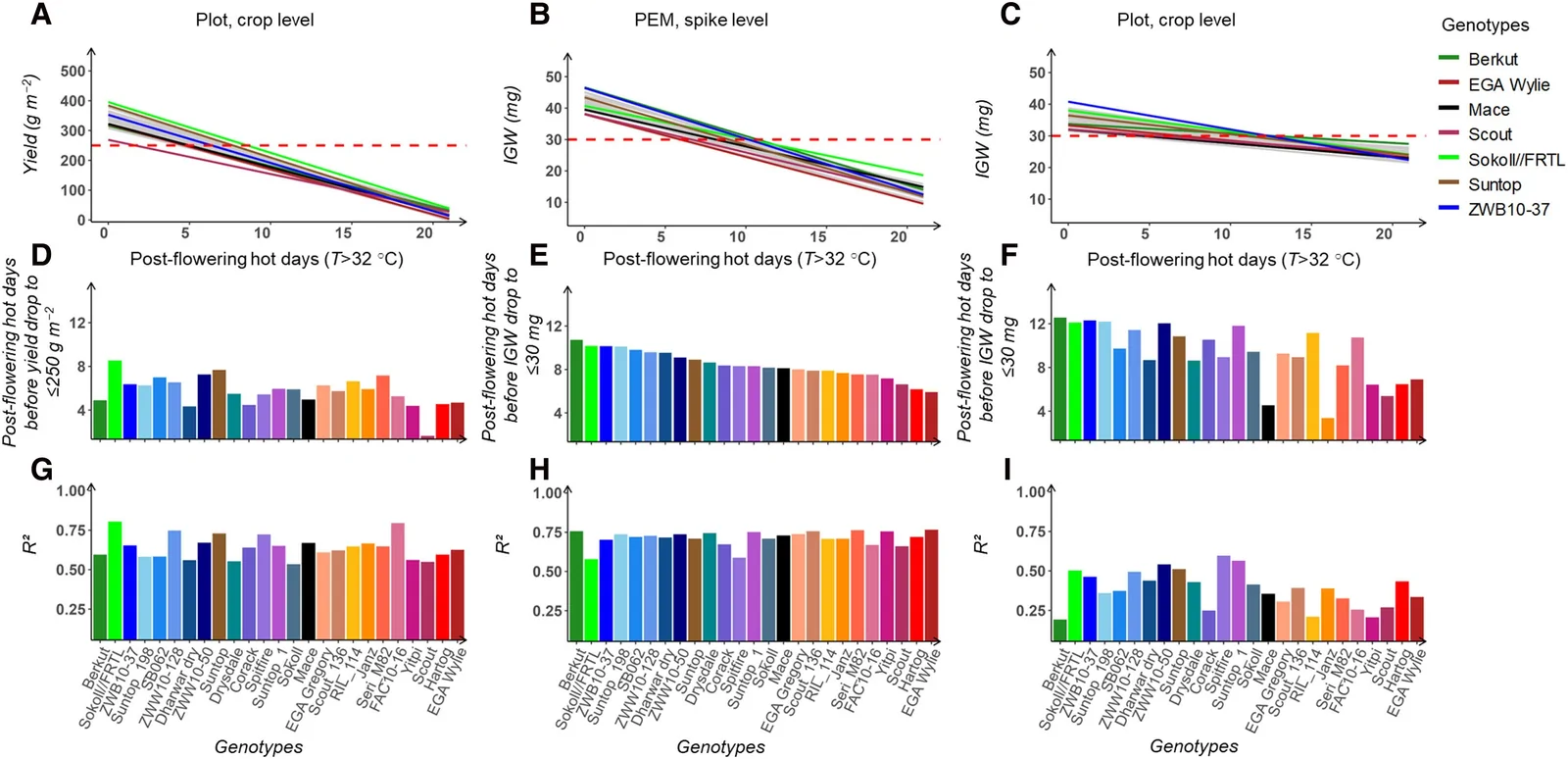
Genotype reaction norms (A–C), genotype tolerance thresholds (D–F), and coefficient of determination from the reaction norms (G–I) for grain yield (A, D, G) and individual grain weight (IGW; B, C, E, F, H, I) in response to post-flowering heat for the 25 studied genotypes in photoperiod-extension method (PEM) trials (spike level; B, E, H) and plot trials (crop level; A, C, D, F, G, I). In (A–C), genotype reaction norms are expressed relative to the number of hot days (*T*>32 °C) occurring between 0 °Cd and 500 °Cd after flowering. Reaction norms for seven selected genotypes are presented in colour including modern benchmark cv. Suntop, Mace, and Scout as well as EGA Wylie, and experimental lines Sokoll//FRTL and ZWB10-37. Red dashed horizontal lines indicate benchmarks, that is 250 g m^−2^ for yield and 30 mg for IGW. In (D–F), bar graphs represent the number of hot days each genotype can endure before dropping below a threshold either 250 g m^−2^ grain yield (D) or 30 mg IGW in the PEM (E) or plot trials (F). In (G–I), bar graphs represent *R*^2^ values from the reaction norms (A–C). In bar graphs (D–I), genotypes are ranked in descending order for the number of post-flowering hot days they can endure before dropping below 30 mg IGW in PEM trials (E). Plots of reaction norms for all 25 genotypes are presented in Supplementary Figs S1, S3. Slope and intercept values of heat response for yield (A), IGW (B), and GPC (C) are presented in Supplementary Tables S3, S4 and Supplementary Fig. S7. Values for the number of hot days that genotypes can endure before reaching 250 g m^−2^ yield threshold, or 30 mg IGW thresholds are presented in Supplementary Table S3.

**Fig. 3.**
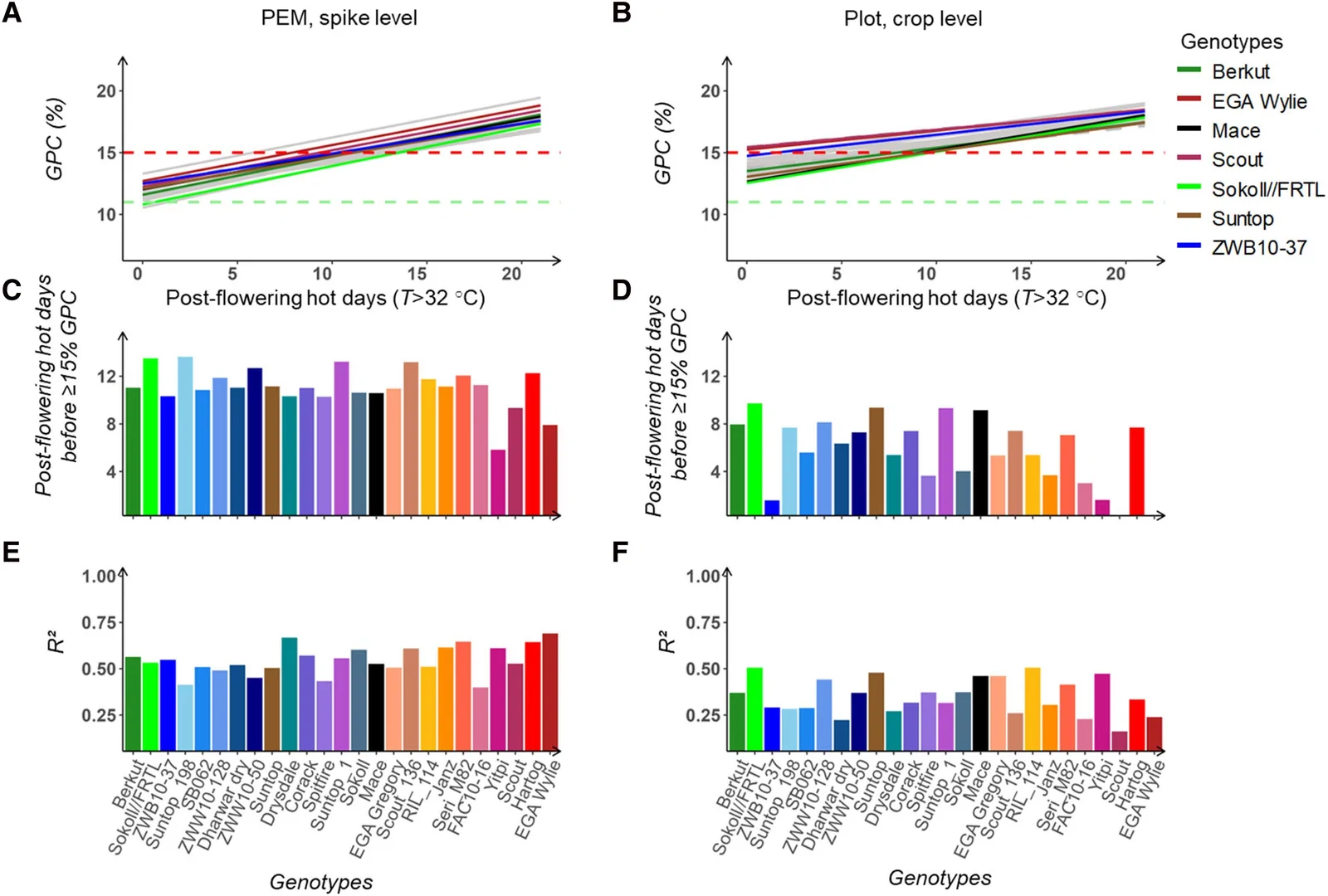
Genotype reaction norms (A, B), genotype tolerance thresholds (C, D) and coefficient of determination from the reaction norms (E, F) for grain protein content (GPC) in response to post-flowering heat for the 25 studied genotypes in photoperiod-extension method (PEM) trials (spike level; A, C, E) and plot trials (crop level; B, D, F). In (A, B), reaction norms are expressed relative to the number of hot days (*T*>32 °C) occurring between 0 °Cd and 500 °Cd after flowering. Reaction norms for seven selected genotypes are presented in colour including modern benchmark cv. Suntop, Mace, and Scout as well as EGA Wylie, and experimental lines Sokoll//FRTL and ZWB10-37. Red and green dashed horizontal lines indicate key GPC thresholds of 15% and 11%, respectively. In (C, D), bar graphs represent the number of hot days each genotype can endure before reaching threshold 15% GPC. In (E, F), bar graphs represent *R*^2^ values of the reaction norms. Plots of reaction norms for all 25 genotypes are presented in Supplementary Fig. S4. Slope and intercept values of GPC heat responses for the 25 studied genotypes are presented in Supplementary Table S4 and Supplementary Fig. S7. Values related to the number of hot days that genotypes can endure before reaching 15% GPC threshold are presented in Supplementary Table S3.

Distinct responses to the number of hot days were observed among genotypes for all studied traits, including yield, IGW, GP, and GPC (Figs 2, 3; Supplementary Figs S1–S5; Supplementary Tables S3, S4). Genotypes with lower plasticity to post-flowering heat for yield and IGW (i.e. response slope closer to 0) typically had lower yield and IGW in non-stress environments (i.e. HET1 or intercept of the linear regression) as highlighted by strongly negative and significant correlations between the slope and intercept of the reaction norms (*P*<0.05, *R*^2^ ranging from 0.57 to 0.83; Supplementary Fig. S6). Similarly, genotypes with lower plasticity for GPC typically had higher IGW in non-stress environments (*R*^2^ of 0.34 and 0.55 for PEM and plot trials, respectively; *P*<0.05). Given these strong correlations between plasticity and trait value in non-stressed environments, the sensitivity (or tolerance) of each genotype for yield, IGW, and GPC was also assessed in terms of the number of post-flowering hot days that a genotype could endure before its yield fell below 250 g m^−2^, IGW fell below 30 mg, and GPC increased to 15% (Figs 2, 3).

For responses of both (i) IGW and (ii) GPC to the number of post-flowering hot days, the plasticity to heat was greater for organ-level measurements in the PEM than for crop-level measurements in plot trials (Supplementary Fig. S7). In addition, these responses had greater coefficients of determination (*R*^2^) and lower *P-*values in the PEM than in plot trials (Figs 2, 3).

Plasticity of grain yield ranged from a yield loss of −11.46 g m^−2^ (Scout) to −17.65 g m^−2^ (ZWW10-50) per post-flowering hot day, corresponding to a variation of 35% across genotypes (Supplementary Fig. S7A). Associated *R*^2^ values ranged from 0.53 to 0.80 among genotypes, implying that heat stress substantially contributed to yield loss of studied genotypes in the tested conditions (Fig. 3G). Genotypes could endure from 1.6 (Scout) to 8.6 (Sokoll//FRTL) post-flowering hot days before yield dropped below 250 g m^−2^, reflecting a 5.4-fold variation among genotypes (Fig. 2D). Genotypes with the highest yield under non-stress HET1 (Supplementary Fig. S8A), that is with higher intercept values (Supplementary Table S4), typically endured a greater number of post-flowering hot days before dropping the 250 g m^−2^ yield benchmark. This was the case of Sokoll//FRTL, Suntop, ZWW10-50, Seri-M82, and SB062, which only dropped to 250 g m^−2^ yield after enduring ≥7 post-flowering hot days.

Plasticity of IGW ranged from a loss of 1.05 mg (Sokoll//FRTL) to 1.61 mg (ZWB10-37) per post-flowering hot day with the PEM (Supplementary Fig. S7B), depicting a variation of 53% across genotypes. In conventional plots, this plasticity was less marked and not as clear, with a loss in IGW only ranging from 0.26 mg (RIL_114) to 0.87 mg (ZWB10-37) per post-flowering hot day (Supplementary Fig. S7C), and with *R*^2^ of 0.19–59 in the plots compared with 0.57–0.76 in the PEM (Fig. 2H, I). On the other hand, the number of post-flowering hot days required to cause IGW to drop to ≤30 mg was greater for the conventional plots than for the PEM, ranging (i) in the PEM, from 5.9 d (EGA Wylie) to 10.7 d (Berkut) (a range of 4.8 hot days), that is an approximate 2-fold difference among genotypes (Fig. 2E), and (ii) in plot trials, from 3.4 d (Janz) to 12.6 d (Berkut) (a range of 9.2 hot days), that is an almost 4-fold difference among genotypes (Fig. 2F).

In both the PEM and plot trials, 15 genotypes endured >8 hot days before dropping ≤30 mg IGW (Fig. 2E, F), and four genotypes (i.e. Berkut, Sokoll/FRTL, Suntop-98, and ZWB10-37) sustained more than 10.0 hot days before reaching this IGW benchmark. Out of these four tolerant genotypes, Sokoll//FRTL appeared as a highly promising line as it also had high yield under non-stress conditions (HET1; Supplementary Fig. S8A) and maintained the highest yield of all genotypes under heat (8.6 hot days before reaching 250 g m^−2^; Fig. 2A). ZWB10-37 also produced a reasonable yield under non-stress HET1 and maintained a high IGW and a relatively high yield under heat (Fig. 2A, B; Supplementary Fig. S8A, C). However, Sokoll//FRTL exhibited superior IGW maintenance, enduring 12.1 post-flowering hot days before its IGW fell below 30 mg, whereas ZWB10-37, despite its initial advantage, experienced a sharper IGW decline after enduring 12.3 post-flowering hot days (Fig. 2B, E; Supplementary Table S4). For comparison, benchmarking cultivars Suntop, Scout, and Mace sustained 10.8, 5.3, and 8.1 hot days, respectively, before reaching the IGW benchmark (Fig. 2B, E) and they also had relatively low IGW in non-stress HET1 (58.4, 50.8, and 51 mg, respectively, compared with 58.4 mg in Sokoll//FRTL and 66.3 mg in ZWB10-37 on average across PEM and plot trials, Supplementary Fig. S8C, D), with an average HET1 yield of 400, 304, and 369 g m^−2^, respectively (compared with 407 g m^−2^ and 378 g m^−2^ for Sokoll//FRTL and ZWB10-37, respectively; Supplementary Fig. S8A).

In terms of protein content, the plasticity of GPC ranged from an increase of 0.23% (Suntop_198) to 0.35% (SB062) per post-flowering hot day with the PEM (Supplementary Fig. S7D) and from 0.14% (Scout) to 0.25% (Mace) in plot trials (Supplementary Fig. S7E). Furthermore, the number of post-flowering hot days required to reach the upper boundary of 15% GPC varied (i) between 5.8 d (Yitpi) and 13.6 d (Suntop_198) with the PEM, i.e. a 2.3-fold genotypic difference (Fig. 3C), and (ii) between 0 d (Scout) and 9.7 d (Sokoll//FRTL) only in plot trials (Fig. 3D). The high-yielding Skoll//FRTL genotype, with high yield in both non-stress and stress conditions (Fig. 2A), had a high level of heat tolerance for yield, IGW, and GPC (Figs 2D, E, 3C). It maintained high IGW under heat stress (Fig. 3C; Supplementary Fig. S7B, F) and relatively low GPC, with a moderate plasticity for GPC (Supplementary Fig. S7D, E) so that it could endure 13.5 and 9.7 post-flowering hot days before exceeding the 15% GPC benchmark in the PEM and plot trials, respectively (Fig. 3C, D; Supplementary Table S3). By contrast, ZWB10-37 exhibited the highest IGW (53.9 mg; Supplementary Fig. S8C) of the studied genotypes, together with a high GPC (12.4%; Supplementary Fig. S8E) in non-stress environments, and had a low GPC plasticity to heat (Supplementary Fig. S7D, E), so that it endured only 10.3 and 1.6 post-flowering hot days before exceeding 15% GPC in the PEM and plot trials, respectively (Fig. 3C, D; Supplementary Table S3).

### An increase in grain protein content was associated with decreased yield and individual grain weight

**Fig. 4.**
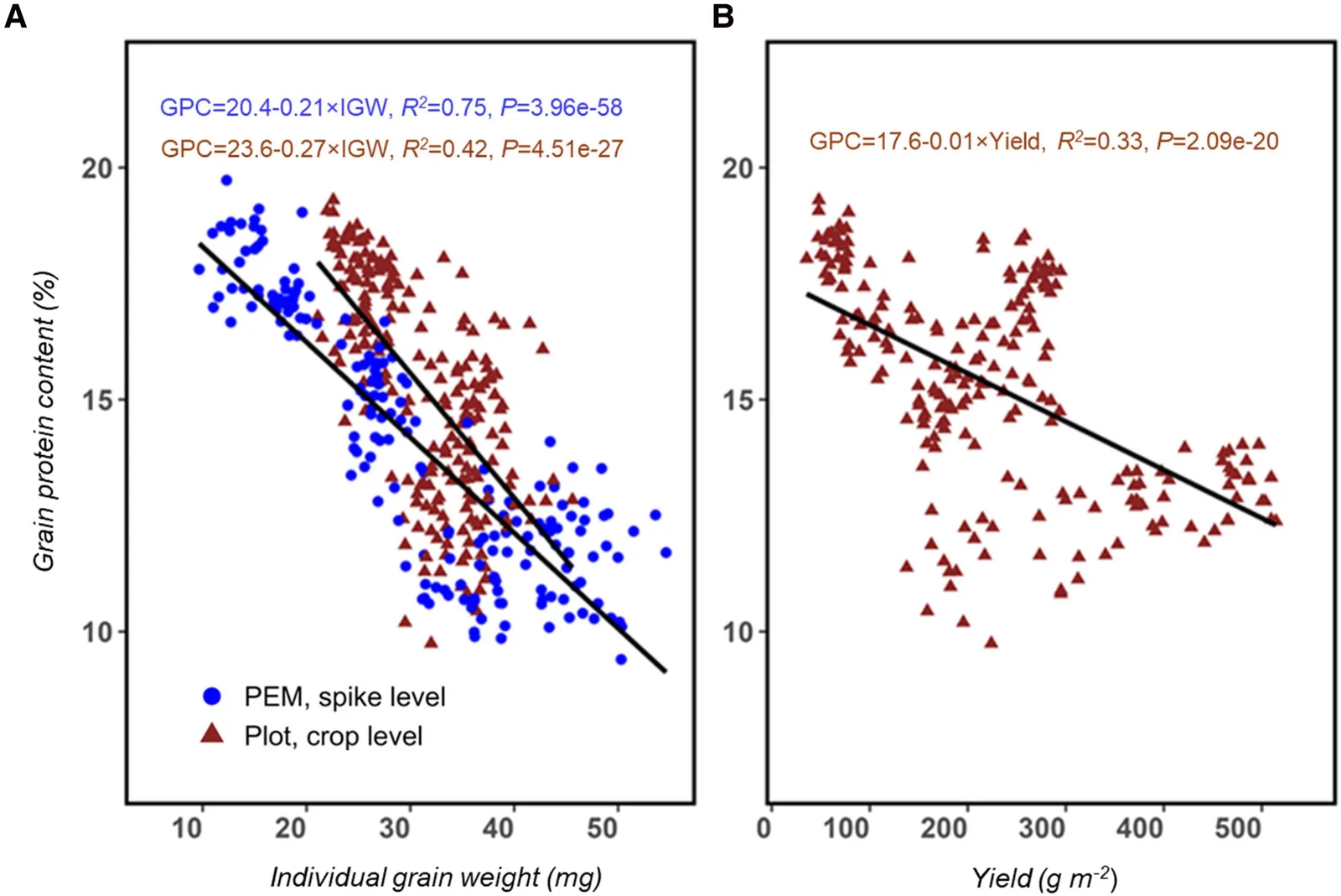
Relationship between grain protein content (GPC) and (A) individual grain weight (IGW) for photoperiod-extension method (PEM) trials (spike level) and plot trials (crop level), and (B) grain yield in plot trials. Each data point represents trial-mean values for a genotype. PEM spike-level data are presented as blue circles and plot crop-level data as brown triangles.

**Fig. 5.**
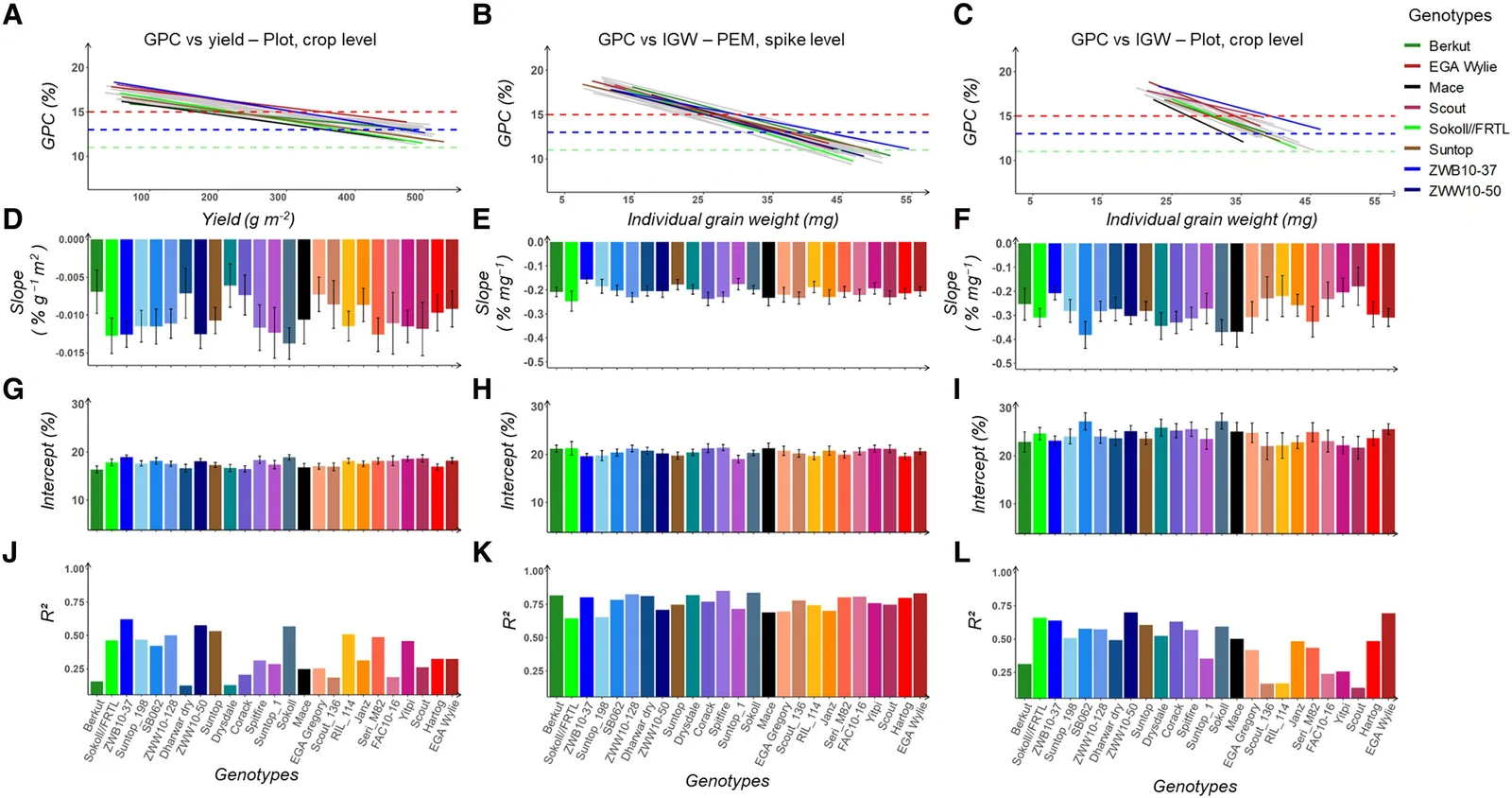
Genotypic variation for response between grain protein content (GPC) and yield (A) and individual grain weight (IGW) (B, C) in photoperiod-extension method (PEM) trials (spike level; B, E, H, K) and plot trials (crop level; C, D, F, G, I, J, L) for the 25 studied genotypes. The slope, intercept and *R*^2^ from each of the linear regressions of GPC vs. either yield or IGW are presented in (D–F), (G–I), and (J–L), respectively. In (A–C), Responses of seven selected genotypes are presented in colour including modern benchmark cv. Suntop, Mace, and Scout, as well as EGA Wylie, and experimental lines Sokoll//FRTL and ZWB10-37. Green, purple, and red dashed horizontal lines correspond to GPC standards for Australian prime hard (APH), i.e. 11.5% and 13%, as well as an upper threshold of 15%. Plots of reaction norms for all 25 genotypes are presented in Supplementary Fig. S9. Slope and intercept values are presented for the 25 studied genotypes in Supplementary Table S4.

GPC was significantly (*P*<0.001) negatively correlated with grain yield, with each 1 g m^−2^ decrease in yield being associated with a 0.01% increase in GPC (*R*^2^=0.34; Fig. 4B). More than 2-fold genotypic variation was observed in the rate of GPC increase per unit of yield loss, ranging from −0.006% g^−1^ m^2^ (Berkut, Drysdale) to −0.013% g^−1^ m^2^ (Sokoll) among genotypes (Fig. 5D; Supplementary Fig. S9). Berkut, the genotype that best maintained IGW above the 30 mg benchmark (Fig. 2B, E), exhibited among the weakest increase in GPC per unit of yield loss (slope=−0.0069% g^−1^ m^2^; Fig. 5D). In contrast, Sokoll (slope of −0.013) and Sokoll//FRTL (−0.012) increased about twice as much GPC per unit of yield loss as Berkut (Fig. 5D).

A strong negative relationship was also observed between IGW and GPC in both the PEM and plot trials, with slope coefficient of −0.21% mg^−1^ and −0.27% mg^−1^, respectively (Fig. 4A). The GPC–IGW relationship was clearer and more consistent in PEM trials (*R*^2^=0.75) than in plot trials (*R*^2^=0.43). Genotypic variation in GPC loss per unit of IGW ranged from −0.24% mg^−1^ (Sokoll//FRTL) to −0.15% mg^−1^ (ZWB10-37) in PEM trials (Fig. 5E) and −0.38% mg^−1^ (SB062) to −0.18% mg^−1^ (Scout) in plot trials (Fig. 5F). The genotype ranking for the GPC–IGW was similar in both the PEM and plot trials, but with greater slopes in plot trials. For instance, Mace lost ∼61% more GPC per unit of IGW gain in plot trials (−0.37% mg^−1^; Fig. 5F) than in spike harvest (−0.23% mg^−1^; Fig. 5E; Supplementary Table S4). The GPC–IGW relationship was much stronger in PEM trials, with *R*^2^>0.6 for all studied genotypes (Fig. 5K), while *R*^2^ ranged from 0.13 to 0.70 in plot trials (Fig. 5L).

## Discussion

Our previous work examined the effects of pre- and post-flowering heat stress on wheat (Ullah *et al.*, 2024). The present study extends this research by using the recently developed PEM (Ullah *et al.*, 2023) to phenotype and discriminate wheat genotypic responses to post-anthesis heat stress under field conditions. Substantial genetic variation was identified among the 25 tested genotypes, with several lines displaying heat tolerance superior to benchmark modern cultivars Suntop, Scout, and Mace, which are widely grown across the northern, southern, and western regions of the Australian wheatbelt, respectively. Results indicate that PEM-based phenotyping can reveal genotypic variation that is not as clearly distinguished using conventional late-sown field trials.

### Benefits of phenotyping spikes with the photoperiod-extension method to screen for heat tolerance

Post-flowering heat stress can severely impair wheat production (Gebeyehou *et al.*, 1982; Thistlethwaite *et al.*, 2015; Telfer *et al.*, 2018; Gerard *et al.*, 2020), yet screening for genotypic tolerance under field conditions remains challenging due to the variable nature of heat episodes and genotypic phenology differences. In the current study, the PEM enabled phenotyping of spikes with synchronized flowering, allowing more precise assessment of heat stress effects on IGW, GP, and GPC than is achievable at the whole-plot level in conventional plots (Figs 2, 3). Across all traits and genotypes, reaction norms derived from PEM trials exhibited higher coefficients of determination (*R*^2^) than those obtained from conventional plot trials, and heat responses were more strongly expressed. For example, IGW loss per post-flowering hot day was approximately three times greater in PEM trials than in plot trials (∼1.5 vs 0.5 mg per day with *T*>32 °C, respectively).

The capacity to closely estimate heat sensitivity with the PEM primarily arises from the ability this method gives to bring phenological synchronization and organ-level precision. The timing of heat stress strongly influences traits like IGW, with the greatest effects occurring during early to mid-grain filling, as shown in control environments (Stone and Nicolas, 1995) and in field conditions (Talukder *et al.*, 2014; Shirdelmoghanloo *et al.*, 2016; Ullah *et al.*, 2024). Heat shocks also affect tillers differently depending on their developmental stage at the time of stress (Chenu and Oudin, 2019). By extending the photoperiod, the PEM allowed alignment of flowering across genotypes, ensuring that spikes were at comparable developmental stages during heat events and thereby reducing confounding effects of phenological variation (Ullah *et al.*, 2023). In the present study, genotypes varied widely in flowering time under natural conditions, ranging from 79 d to 110 d after sowing in standard sowings (s1), and from 57 d to 78 d in late sowings (s2). Previous approaches to reduce phenological variation such as grouping genotypes by maturity class or varying sowing dates (Frederiks *et al.*, 2012) often expose genotypes to different radiation, temperature, and rainfall regimes, complicating interpretation of stress responses (Chenu *et al.*, 2013; Ullah *et al.*, 2023). Although the PEM itself may influence genotype responses by differentially altering phenology and associated traits (e.g. source–sink relationships), genotype rankings have been shown to be highly consistent within heat environment types (HETs; Ullah *et al.*, 2023), indicating that the method robustly captures heat effects.

The PEM can be applied in single trials or expanded to multi-environment trials, enabling the establishment of genotype-specific reaction norms and the identification of genotype ‘meta-mechanisms’ (Tardieu, 2003). In the current study, all genotypes were included in every trial using a balanced design, ensuring exposure to comparable environments and enabling robust comparison of reaction norms. In contrast, when unbalanced designs are used, reaction norms should preferably be developed for traits expressed relative to integrative environmental variables such as cumulative radiation or the photothermal quotient.

Overall, the PEM, combined with organ-level phenotyping, proved highly effective in uncovering genotypic differences that are often masked in conventional field plots. Although demonstrated here on 25 genotypes, the method could readily be upscaled, for example by focusing on synchronized quadrats rather than individual spikes (Ullah *et al.*, 2023) and by integrating high-throughput technologies such as proximal or UAV-based sensing (Chapman *et al.*, 2014; Potgieter *et al.*, 2018). Moreover, while single-row plantings are sufficient for heat-stress studies, small PEM-managed plots could be used to investigate combined heat and drought stress, as these stresses commonly co-occur in production environments (Lake *et al.*, 2025) and trigger intricate interactions affecting genotype responses (Vadez *et al.*, 2024; Gawinowski *et al.*, 2025).

### A wide range of reaction norms in response to heat, with some interesting phenotypes

Post-anthesis heatwaves cause substantial yield losses even under well-watered conditions (Fig. 1A). On average, yield of field plots declined by 15.1 g m^−2^ (3.5%) for each additional post-flowering day with a temperature >32 °C (between 0 °Cd and 500 °Cd after flowering). This is similar to the 16.1 g m^−2^ per hot day (*T*>30 °C) between 100 and 600 °Cd post-anthesis previously observed in field conditions in southern Australia (Telfer *et al.*, 2018). Across studies for well-watered wheat crops, reported yield losses range widely, between 3% and 18% per °C in post-flowering temperature above optimum (Mondal *et al.*, 2013; Ullah, 2018; He *et al.*, 2022), as others environmental factors impact crop response and can interact with heat effects (Slafer *et al.*, 2023; Gawinowski *et al.*, 2025).

Unlike earlier studies that compared stressed and non-stressed environments categorically (e.g. Thistlethwaite *et al.*, 2020), this study quantified continuous reaction norms to heat. Plasticity was expressed for traits like yield, IGW, and GPC as a function of the number of hot days (*T*>32 °C) experienced between 0 °Cd and 500 °Cd after flowering. A wide range of variations was observed, with plasticity varying among genotypes from −13.1 g m^−2^ to −17.5 g m^−2^ per hot day for yield (i.e. ∼34% variation), from 1.05 mg to 1.61 mg per hot day for IGW (53% variation) and from 0.23% to 0.35% per hot day for GPC (52% variation). To put this in perspective, on average, for 2 d of heatwave, a sensitive genotype might lose 34 g m^−2^ (0.34 t ha^−1^), while a tolerant one would only lose 26 g m^−2^ (0.26 t ha^−1^).

Strong correlations were observed between reaction norm slopes (heat sensitivity) and intercepts (trait values under non-stress). For instance, genotypes best sustaining IGW under heat were typically those with the lowest IGW under non-stress conditions (Supplementary Fig. S8). To better reflect production relevance, new heat-tolerance metrics were introduced, defined as the number of post-flowering hot days a genotype can endure before (i) its yield dropped to 250 g m^−2^ (a threshold chosen for Australian wheat), (ii) its IGW dropped to 30 mg, and (iii) its GPC increased to an upper threshold of 15%. These metrics revealed large genotypic variation, exceeding 5-fold for yield thresholds and approaching 2-fold for IGW and GPC thresholds (Figs 2, 3; Supplementary Table S3).

Underlying traits involved in observed heat tolerance would be interesting to explore with the PEM. These traits likely include delayed senescence (Lopes *et al.*, 2012; Pinto *et al.*, 2016; Yahya *et al.*, 2022; Ullah *et al.*, 2026), increased stem carbohydrate remobilization (Blum *et al.*, 1994; Clifton *et al.*, 2025), and maintenance of grain cell division and expansion (Girousse *et al.*, 2021). For instance, genotype SB062 consistently maintained grain size (Fig. 3E) and leaf greenness after exposure to heat (Ullah and Chenu, 2019; Ullah *et al.*, 2026). This line is also known to have a high concentration of stem water soluble carbohydrates at anthesis (Dreccer *et al.*, 2009), which can be valuable for remobilization to grains under stress.

### Implications for the industry

Post-flowering heatwaves have increased in frequency in recent decades (Ababaei and Chenu, 2020) and are projected to intensify under climate change (Collins and Chenu, 2021). In Australia, the number of hot days (*T*>32 °C) between August and November increased by 2.6 d per decade between 1985 and 2017 (Ababaei and Chenu, 2020). If considering this rate for the grain filling period, grains of the modern benchmark Suntop, which lose 1.48 mg per extra hot post-flowering day, would lose 7.7 mg each in two decades (i.e. 16% compared with 47.7 mg in non-stressful HET1 environments), while grains of the tolerant genotype Sokoll//FRTL would only lose 5.7 mg, i.e. 26% less IGW loss. These findings highlight the importance of combining (i) agronomic adaptation as previously recommended (e.g. Flohr *et al.*, 2018; Collins and Chenu, 2021) and as already currently occurring in some regions [e.g. with farmers sowing their crops earlier in South Australia (Flohr *et al.*, 2018) to avoid terminal heat and drought (Chenu *et al.*, 2013)] and (ii) genetic improvement through breeding, in particular given the existing level of genetic variability for heat tolerance (as clearly evidenced in the current study).

Several heat-tolerant lines identified here (Figs 2, 3) demonstrated superior performance under both stress and non-stress conditions, making them strong candidates for breeding programs targeting heat-prone region. For instance, Sokoll//FRTL endured 8.6 hot post-flowering days (*T*>32 °C within 0–500 °Cd post-flowering) before its yield dropped below 250 g m^−2^ (compared with 7.7, 1.6, and 5.0 d for benchmark modern cultivars Suntop, Scout, and Mace). Further, in non-stress conditions, Sokoll//FRTL outperformed or had similar yield (407 g m^−2^) to benchmark cv. Suntop, Scout, and Mace (400, 304, and 369 g m^−2^, respectively; Supplementary Fig. S8A). Sokoll//FRTL also sustained IGW above 30 mg for over 10.0 post-flowering hot days, showing again superior adaptation to heat than benchmark cultivars that only sustained such IGW for 8.9, 6.6, and 8.1 hot days, respectively (Fig. 2E, F). Alongside superior IGW retention, Sokoll//FRTL showed high GPC stability, with a GPC remaining below the 15% upper threshold for up to 13.5 post-flowering hot days compared with 11.2, 9.4, and 10.6 d for Suntop, Scout, and Mace, respectively (Fig. 3C). Overall, with its high performance under non-stressful environments and its capacity to sustain grain size, yield, and protein content in stressful conditions, Sokoll//FRTL appears a valuable candidate for breeding programmes aiming at enhancing wheat productivity and resilience in regions prone to post-flowering heatwaves (Ababaei and Chenu, 2020). Looking ahead, as discussed previously, the potential to substantially increase the PEM throughput could enable screening of diverse populations, facilitating the identification of many more heat-tolerant genotypes for industrial benefit.

Heat stress during grain filling not only substantially impacts grain size and yield but is also deleterious for end-use grain quality (Balla *et al.*, 2009; Dias and Lidon, 2009; Cosentino *et al.*, 2018). The current study assessed genotypes in terms of their heat response for average grain size and GPC, but further analysis would be required to characterize them for other important quality factors such as screenings and glutenin-to-gliadin ratio. Previously, high GPC level under stress, as observed in the current study (Fig. 4A, B), has been associated with alterations in protein composition, such as decrease in the glutenin-to-gliadin ratio, which negatively affects dough rheology and end-use properties in bread wheat (Daniel and Triboi, 2000; Altenbach *et al.*, 2003; Chunduri *et al.*, 2021). In addition, smaller and shrunken grain, as observed in some of the studied genotypes and trials here, is generally reported to contain less starch and can present irregular grain size distributions, directly impairing flour extraction efficiency by increasing bran content and decreasing flour yield during milling operations (Labuschagne *et al.*, 2009; Cosentino *et al.*, 2018).

Finally, the quality reaction norms identified with the PEM for genotypes with contrasted heat-tolerance levels provide valuable insights for improving crop models, refining their heat-response parameters, and supporting GxExM adaptation strategies as well as genotype selection for climate-resilient wheat production (Chenu *et al.*, 2017).

## Conclusion

Tolerance to heat was assessed for 25 genotypes by evaluating their reaction norms for yield, IGW, and GPC in response to the number of post-flowering hot days (*T*>32 °C; 0–500 °Cd after flowering). Using the PEM to match developmental stages and focusing on organ-level phenotyping substantially improved phenotyping accuracy and resolution of reaction norms compared with crop-level results from conventional field plots. For each studied trait, the plasticity to heat (slope of the reaction norm) of tested genotypes was negatively correlated with their trait value in non-stress environments (intercept). Targeting agronomic relevance, heat tolerance was also evaluated in terms of the stress level a genotype could endure before reaching a meaningful threshold for the Australian grain industry. Genotypes were characterized for the number of post-flowering hot days they could sustain before reaching IGW of 30 mg, yield of 250 g m^−2^, and an upper limit of 15% GPC. More than 2-fold variations were found for these traits (number of days to reach the thresholds), with heat tolerance of some genotypes extending well beyond the level of tested benchmarking modern Australian cultivars.

The results of the study, with quantitative reaction norms characterizing quite clearly (high *R*^2^) the 25 studied genotypes, open avenues for crop modelling studies to (i) refine heat responses within models and (ii) use meaningful genotypic parametrization to propose realistic adaptation strategies in the face of climate change.

Finally, upscaling the high-resolution phenotyping method used in this study to allow high throughput screening of large numbers of genotypes offers considerable potential to accelerate the development of wheat cultivars capable of sustaining yield and maintaining grain quality under increasingly frequent post-flowering heatwaves.

## Supplementary Material

erag188_Supplementary_Data

## Data Availability

All primary data to support the findings of this study are openly available at https://doi.org/10.48610/e38b8c9 (Yahya *et al.*, 2026).

## Supplementary data

The following supplementary data are available at *JXB* online.

Fig. S1. Yield response to the number of post-flowering hot days for the 25 studied genotypes.

Fig. S2. Grain number response to the number of pre-flowering hot days for the 25 studied genotypes.

Fig. S3. Response of individual grain weight to the number of post-flowering hot days for the 25 studied genotypes in PEM and plot trials.

Fig. S4. Grain protein content response to the number of post-flowering hot days for the 25 studied genotypes in PEM and plot trials.

Fig. S5. Response of grain number to pre-flowering hot days for mean-trial values, and each of the 25 studied genotypes in plot trials.

Fig. S6. Relationships between the slope and intercept values of the reaction norms of yield, individual grain weight, and grain protein content in response to the number of post-flowering hot days in PEM and plot trials.

Fig. S7. Heat sensitivity of yield, individual grain weight, and grain protein content in PEM and plot trials.

Fig. S8. Grain yield, grain number, individual grain weight, and grain protein content for the 25 studied genotypes in PEM and plot trials with no or limited heat stress (i.e. HET1).

Fig. S9. Relationship between grain protein content and individual grain weight in PEM and plot trials (crop level), and yield in plot trials.

Table S1. Characteristics of the 25 wheat genotypes grown in the PEM and conventional field trials.

Table S2. Analysis of variance for grain yield, grain number, individual grain weight, grain protein amount and grain protein content of the 25 studied wheat genotypes grown with (i) the photoperiod-extension method (PEM), and (ii) conventional plots across trials.

Table S3. Number of post-flowering hot days that genotypes could endure before reaching thresholds of 250 g m^−2^ grain yield, 30 mg IGW, and 15% GPC in PEM and plot trials.

Table S4. Genotypic values of the slopes and intercepts from (i) the linear model of yield, individual grain weight (IGW), or grain protein content in response to heat stress expressed as the number of hot days occurring between 0 °Cd and 500 °Cd after flowering, and (ii) the linear relationship between GPC vs yield or IGW, for the 25 studied genotypes in PEM and plot trials.
