# Supplementary material for: Improving wheat tolerance to post-flowering heat using matched development stages and field-based reaction norms
Source: J Exp Bot. 2026 Apr 17;77(17):5791–806. doi: 10.1093/jxb/erag188 (PMC13577760; doi:10.1093/jxb/erag188)
Supplement: Supplementary file 1 [file erag188_supplementary_data.pdf]

Supplementary information

Improving wheat tolerance to post-flowering heat using matched development stages and field-based reaction norms

Muhammad Yahya[1,2], Najeeb Ullah [3], Jack Christopher[1], Daniel Cozzolino[2] , Vincent Mellor[5], Glen Fox[4], Estelle Kuhn[6] and Karine Chenu[1,7,8]*

[1] The University of Queensland, Queensland Alliance for Agriculture and Food Innovation (QAAFI), Building 8115, 5391 Warrego Hwy, Gatton, Queensland 4343, Australia.

[2] The University of Queensland, Queensland Alliance for Agriculture and Food Innovation (QAAFI), Brisbane, St Lucia, Queensland 4072, Australia.

[3] Agriculture Research Station, office of VP For Research and Graduate Studies, Qatar University, Doha, Qatar.

[4] University of California Davis, Department of Food Science and Technology, Davis, CA 95616, USA.

[5] The University of Queensland, School of Agriculture and Food Sustainability, 5391 Warrego Hwy, Gatton, Queensland 4343, Australia.

[6] Université Paris-Saclay, INRAE, MaIAGE, 78350, Jouy-en-Josas, France.

[7] ARC Training Centre in Predictive Breeding, The University of Queensland, St Lucia, QLD 4072, Australia.

[8] ARC Hub for Engineering Plants to Replace Fossil Carbon, The University of Queensland, St Lucia, QLD 4072, Australia.

* Corresponding author: karine.chenu@uq.edu.au

Supplementary Table S1. Characteristics of the 25 wheat genotypes grown in the PEM and conventional field trials.

Genotype	Type	Pedigree	Characteristics	Reference
Berkut	Cultivar	Irena/Baviacora-M-92//Pastor	Heat tolerance	Thistlethwaite et al., 2020
Corack	Cultivar	MACHETE/84W129-504*2/5/VICAM S 71//CIANO F 67/SIETE CERROS/ 3/KAL/BB/4/TM56	Australian Premium White (APW) variety	GRDC, 2018
Dharwar Dry	Cultivar	DWR39/C306//HD2189	Drought tolerant, stay-green phenotype	Manschadi et al., 2008
Drysdale	Cultivar	Hartog*3/Quarrion	Superior transpiration efficiency	Condon et al., 2004<br>Richards et al., 2006<br>Rebetzke et al., 2009<br>Fletcher et al., 2018
EGA Gregory	Cultivar	Pelsart/2*Batavia	Long-season elite cultivar with broad adaptation	
EGA Wylie	Cultivar	INIA F 66 / GAMUT// COOK/3/JUPATECO F 73/TR 59	Disease resistance	Zheng et al., 2014
FAC10-16	Elite breeding line	10CB-F/W234	Disease resistance	Dinglasan et al., 2016
Hartog	Cultivar	Vicam 71//Ciano 's'/Siete Cerros/3/Kalyansona/Bluebird	Drought sensitive, senescent phenotype	Christopher et al., 2008
Janz	Cultivar	3-AG-3/4*CONDOR//COOK	Drought sensitive, senescent phenotype	Christopher et al., 2008
Mace	Cultivar	Wyalkatchem/Stylet//Wyalkatchem	Benchmark cultivar for yield across southern Australian cropping regions	GRDC, 2018
RIL114	Experimental line	UQ01484/RSY10//H45	Pre-harvest sprouting tolerance	Hickey et al., 2009
SB062	Experimental line	Seri-M82/Babax	Heat tolerant, high stem water soluble carbohydrates	Dreccer et al., 2009<br>Olivares-Villegas et al., 2007<br>Rattey et al., 2009<br>Ullah and Chenu, 2019
Scout	Cultivar	Sunstate/QH71-6//Yitpi	Elite cultivar with broad adaptation	
Scout-136	Elite breeding line	Scout/RIL114	Drought sensitive, senescent phenotype	Christopher et al., 2021
Seri-M82	Elite breeding line	Kavkaz/4/Saric F 70///Lerma Rojo 64A/Inia F66//Inia F66/Yecora F70/5/II-26992	Dense root system, stay-green phenotype	Christopher et al., 2008<br>Olivares-Villegas et al., 2007
Sokoll	Cultivar	Pastor/3/Altar84/AE.SQ (TR.TA)//OPATA-M-85	Heat tolerant	Thistlethwaite et al., 2020
Sokoll//FRTL	Elite breeding line	SOKOLL//FRTL/2*PIFED	Heat tolerant	Thistlethwaite et al., 2020
Spitfire	Cultivar	Drysdale/Kukri	Heat tolerant, high grain protein content	
Suntop	Cultivar	Sunco/2*Pastor//SUN436E	Elite cultivar with broad adaptation; superior transpiration efficiency	Fletcher et al., 2018<br>Collins et al., 2021

Suntop_1	Experimental line	Suntop /Dharwar Dry	Drought tolerant, stay-green phenotype	Christopher et al., 2021
Suntop _198	Experimental line	Suntop/SB062	Drought tolerant, stay-green phenotype	Christopher et al., 2021
Yitpi	Cultivar	Condor/Gabo	Long coleoptile, very susceptible to yellow spot	
ZWB10-37	Experimental line	Tacupeto F2001/Brambling//Kiritati	High yield in CIMMYT-Australia-ICARDA Germplasm Evaluation	Dinglasan et al., 2016
ZWW10-128	Experimental line	ESDA/KKTS	High yield in CIMMYT-Australia-ICARDA Germplasm Evaluation	Dinglasan et al., 2016
ZWW10-50	Experimental line	Onix/4/Milan/Kauz//Prinia/3/BAV92	High yield in the CIMMYT-Australia-ICARDA Germplasm Evaluation	Dinglasan et al., 2016

Supplementary Table S2. Analysis of variance for grain yield (g m^{-2}), grain number (m^{-2}), individual grain weight (IGW, mg), grain protein amount (GP) (mg $grain^{-1}$) and grain protein content (GPC, %) of the 25 studied wheat genotypes grown with (i) the photoperiod-extension method (PEM), and (ii) conventional plots across trials. *, **, *** indicate significant differences for 0.05, 0.01, 0.001 levels, respectively; NS corresponds to non-significant effects; and NA indicates where data were not available.

Trial type - Harvest type		Yield	Grain number	IGW	GP	GPC
PEM - Spike level						
	Location-Year			***	***	***
	Sowing			***	***	***
	Genotype			***	***	***
	Location-Year:Sowing	NA	NA	***	***	***
	Location-Year:Genotype			***	*	**
	Sowing:Genotype			*	NS	NS
	Location-Year:Sowing:Genotype			NS	NS	**
Plots - Crop level						
	Location-Year	***	***	***	***	***
	Sowing	***	***	***	***	NS
	Genotypes	***	***	***	***	***
	Location-Year:Sowing	***	***	***	***	***
	Location-Year:Genotype	***	***	***	***	***
	Sowing:Genotype	***	***	**	*	**
	Location-Year:Sowing:Genotype	***	***	***	***	***

Supplementary Table S3. Number of post-flowering hot days (T>32 °C) that genotypes could endure before reaching thresholds of 250 g m^{-2} grain yield, 30 mg IGW, and 15% GPC in PEM trials (spike level) and plot trials (crop level). Values are colour coded from most beneficial (green) to most detrimental (red).

	PEM, spike level		Plot, crop level		
**Genotype**	**Number of hot days to reach IGW ≤30 mg**	**Number of hot days to reach GPC ≥15 %**	**Number of hot days to reach yield ≤250 g m^{-2}**	**Number of hot days to reach IGW ≤30 mg**	**Number of hot days to reach GPC ≥15%**
Berkut	10.7	11.0	4.9	12.6	7.9
Sokoll//FRTL	10.2	13.5	8.6	12.1	9.7
ZWB10-37	10.2	10.3	6.4	12.3	1.6
Suntop_198	10.1	13.6	6.3	12.2	7.7
SB062	9.8	10.8	7	9.7	5.6
ZWW10-128	9.6	11.9	6.6	11.4	8.1
Dharwar dry	9.5	11.1	4.4	8.7	6.4
ZWW10-50	9.1	12.7	7.2	12.0	7.3
Suntop	8.9	11.2	7.7	10.8	9.4
Drysdale	8.6	10.3	5.5	8.6	5.4
Corack	8.4	11.0	4.5	10.5	7.4
Spitfire	8.3	10.3	5.4	8.9	3.7
Suntop_1	8.3	13.2	6.0	11.8	9.3
Sokoll	8.2	10.6	5.9	9.4	4.0
Mace	8.1	10.6	5.0	4.5	9.1
EGA Gregory	8.0	11	6.3	9.3	5.4
Scout_136	7.9	13.2	5.7	9.0	7.4
RIL_114	7.9	11.7	6.6	11.2	5.4
Janz	7.7	11.1	5.9	3.4	3.7
Seri_M82	7.6	12.1	7.2	8.2	7.1
FAC10-16	7.5	11.3	5.3	10.7	3.0
Yitpi	7.2	5.8	4.4	6.4	1.6
Scout	6.6	9.4	1.6	5.4	-2.9
Hartog	6.2	12.3	4.6	6.5	7.7
EGA Wylie	5.9	7.9	4.7	6.9	-1.5

Supplementary Table S4. Genotypic values of the slopes and intercepts from (i) the linear model of yield (g m^{-2}), individual grain weight (IGW, mg), or grain protein content (GPC, %) in response to heat stress expressed as the number of hot days (T>32 ºC) occurring between 0 and 500 ºCd after flowering, and (ii) the linear relationship between GPC vs. yield or IGW, for the 25 studied genotypes in PEM and plot trials. Slope and intercept values are colour coded from most beneficial (green) to most detrimental (red).

Genotype	PEM Spike level						Plot Crop level									
	IGW vs Heat		GPC vs Heat		GPC vs IGW		Yield vs Heat		IGW vs Heat		GPC vs Heat		GPC vs Yield		GPC vs IGW	
	Slope	Intercept	Slope	Intercept	Slope	Intercept	Slope	Intercept	Slope	Intercept	Slope	Intercept	Slope	Intercept	Slope	Intercept
Berkut	-1.7	47.7	0.32	11.5	-0.21	21.2	-13.8	321	-0.5	35.4	0.18	13.5	-0.007	16.4	-0.25	22.9
Sokoll//FRTL	-1	40.7	0.32	10.8	-0.25	21.3	-16.9	397	-0.7	38.0	0.26	12.6	-0.013	17.8	-0.31	24.6
ZWB10-37	-1.6	47.4	0.21	12.4	-0.16	19.6	-16.1	354	-0.8	40.8	0.16	14.8	-0.013	18.9	-0.21	23.2
Suntop_198	-1.5	44.5	0.15	12.5	-0.19	19.8	-14.2	337	-0.5	35.9	0.18	13.7	-0.011	17.6	-0.28	24
SB062	-1.5	46.5	0.41	10.7	-0.2	20.4	-16.4	360	-0.4	34.6	0.22	13.6	-0.012	18.2	-0.38	27.2
ZWW10-128	-1.6	45.6	0.3	11.3	-0.23	21.2	-18.4	373	-0.7	38.0	0.26	12.8	-0.011	17.6	-0.28	24
Dharwar dry	-1.4	45.5	0.27	12.3	-0.2	20.8	-13.5	304	-0.6	35.6	0.18	14	-0.007	16.6	-0.27	23.6
ZWW10-50	-1.2	40.9	0.25	11.7	-0.21	20.2	-16.2	364	-0.8	39.4	0.2	13.5	-0.013	18.1	-0.3	25.1
Suntop	-1.5	43.8	0.29	11.8	-0.18	19.8	-17.9	384	-0.7	37.3	0.23	12.8	-0.011	17.3	-0.28	23.6
Drysdale	-1.5	43.0	0.32	11.9	-0.2	20.4	-16.5	349	-0.5	34.7	0.19	13.9	-0.006	16.7	-0.34	25.9
Corack	-1.2	41.3	0.27	11.9	-0.24	21.3	-15.3	321	-0.4	34.2	0.2	13.4	-0.007	16.5	-0.33	25.3
Spitfire	-1.1	40.1	0.15	13.5	-0.23	21.4	-15.9	345	-0.7	36.3	0.18	14.3	-0.012	18.4	-0.31	25.6
Suntop_1	-1.4	42.0	0.27	11.6	-0.18	19	-14.8	339	-0.7	37.7	0.25	12.6	-0.012	17.4	-0.27	23.5
Sokoll	-1.3	40.4	0.26	12.1	-0.2	20.3	-14.7	338	-0.6	35.3	0.27	13.6	-0.014	18.9	-0.37	27.2
Mace	-1.1	39.9	0.29	12.1	-0.23	21.3	-16.5	347	-0.5	32.8	0.3	12.2	-0.011	16.8	-0.37	25.1
EGA Gregory	-1.2	40.1	0.27	12.1	-0.22	20.8	-14.9	343	-0.3	32.9	0.18	13.9	-0.007	17	-0.31	24.8
Scout_136	-1.5	42.1	0.36	10.3	-0.23	20.2	-15.1	336	-0.3	32.9	0.21	13.4	-0.009	16.9	-0.23	22
RIL_114	-1.2	40.0	0.25	12	-0.19	19.7	-16	359	-0.2	32.9	0.22	13.6	-0.011	18.1	-0.22	22.1
Janz	-1.1	38.6	0.31	11.6	-0.23	20.8	-15.6	344	-0.5	31.5	0.13	14.6	-0.009	17.6	-0.26	22.8
Seri_M82	-1.4	41.2	0.27	11.7	-0.21	20	-16.7	376	-0.4	33.6	0.24	13.3	-0.013	18.2	-0.33	25
FAC10-16	-1.3	40.4	0.18	12.7	-0.22	20.7	-13.5	324	-0.5	34.4	0.16	14.8	-0.011	18.2	-0.23	23.1
Yitpi	-1.5	41.6	0.36	12.7	-0.19	21.2	-13.8	310	-0.3	32.5	0.23	14.4	-0.012	18.6	-0.2	22.2
Scout	-1.4	41.1	0.29	12.2	-0.23	21.2	-13.2	290	-0.4	32.4	0.13	15.4	-0.012	18.7	-0.18	21.7
Hartog	-1.3	38.9	0.28	11.4	-0.21	19.6	-16.1	330	-0.6	33.5	0.16	13.6	-0.010	16.9	-0.3	23.7
EGA Wylie	-1.3	37.8	0.34	12.5	-0.21	20.6	-14.4	317	-0.5	33.0	0.18	15.1	-0.009	18.2	-0.31	25.6

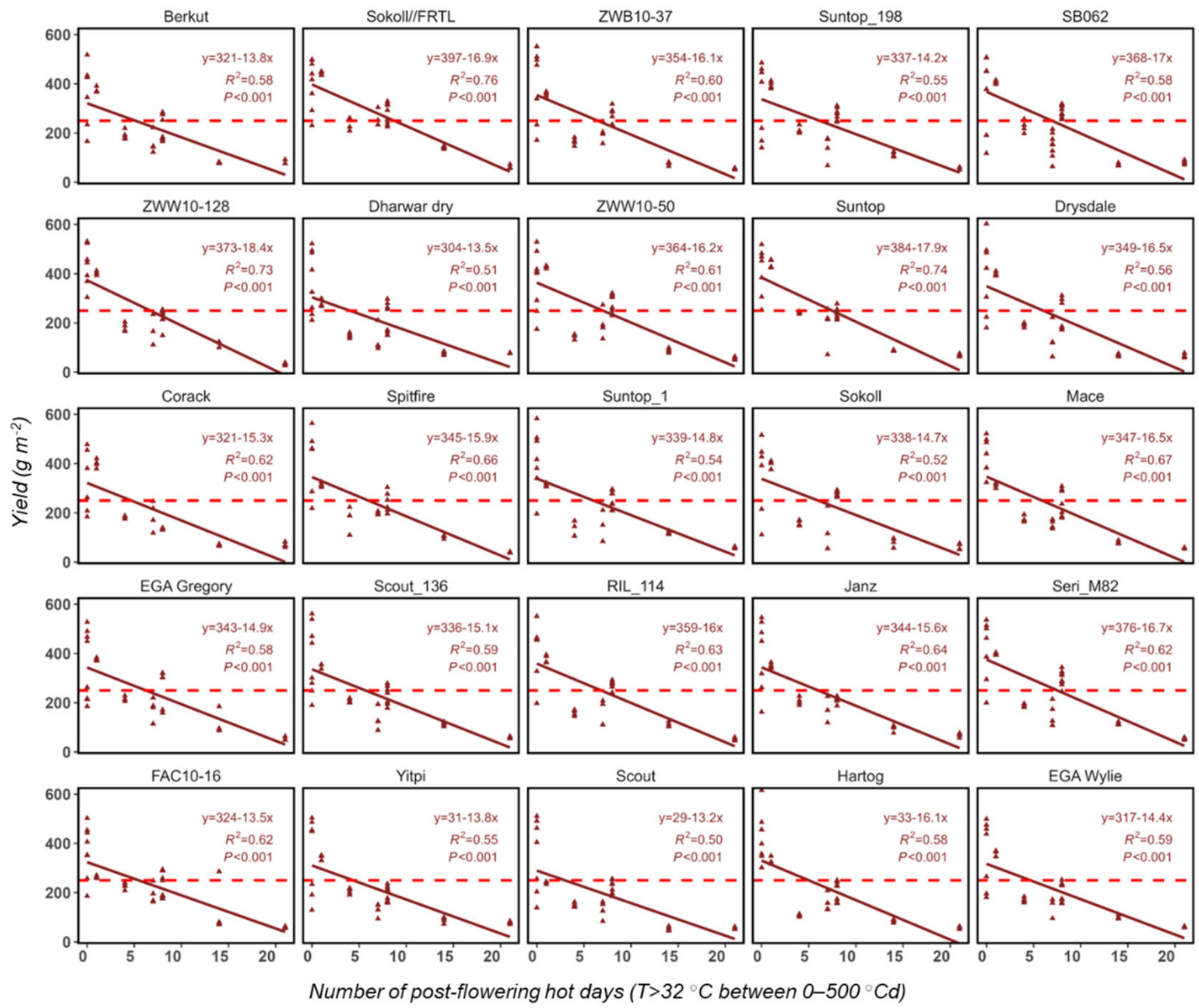

Supplementary Figure S1. Yield response to post-flowering hot days for the 25 studied genotypes. Reaction norms are expressed relative to the number of hot days (T>32 °C) occurring between 0 and 500 °Cd after flowering. Each point corresponds to raw data of each plot for each genotype in each trial. The red dashed horizontal line corresponds to the 250 g m^{-2} yield threshold.

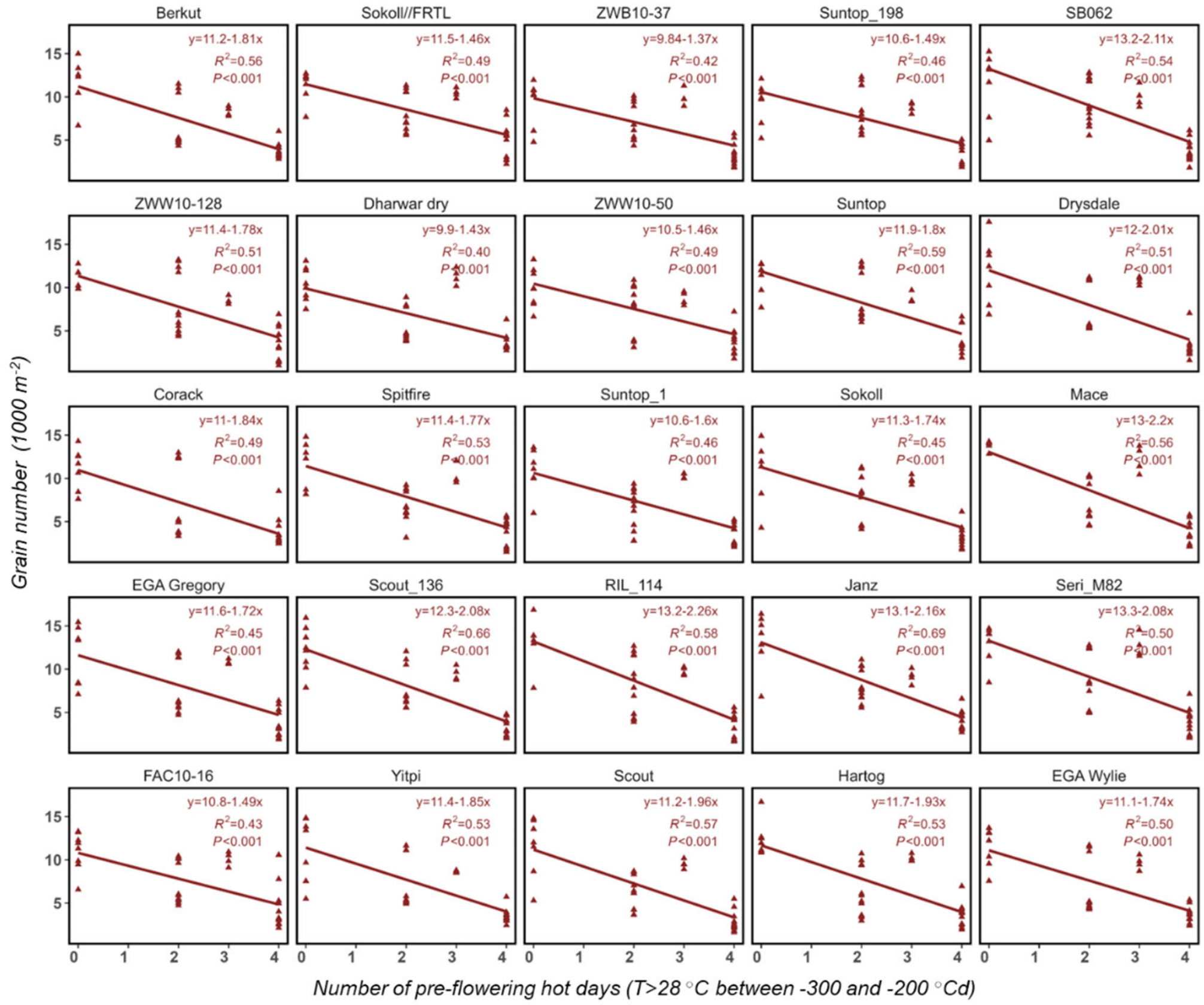

Supplementary Figure S2. Grain number response to pre-flowering hot days for the 25 studied genotypes. Reaction norms are expressed relative to the number of hot days (T>28 °C) occurring between -300 and -200 °Cd before flowering. Each point corresponds to raw data of each plot for each genotype in each trial.

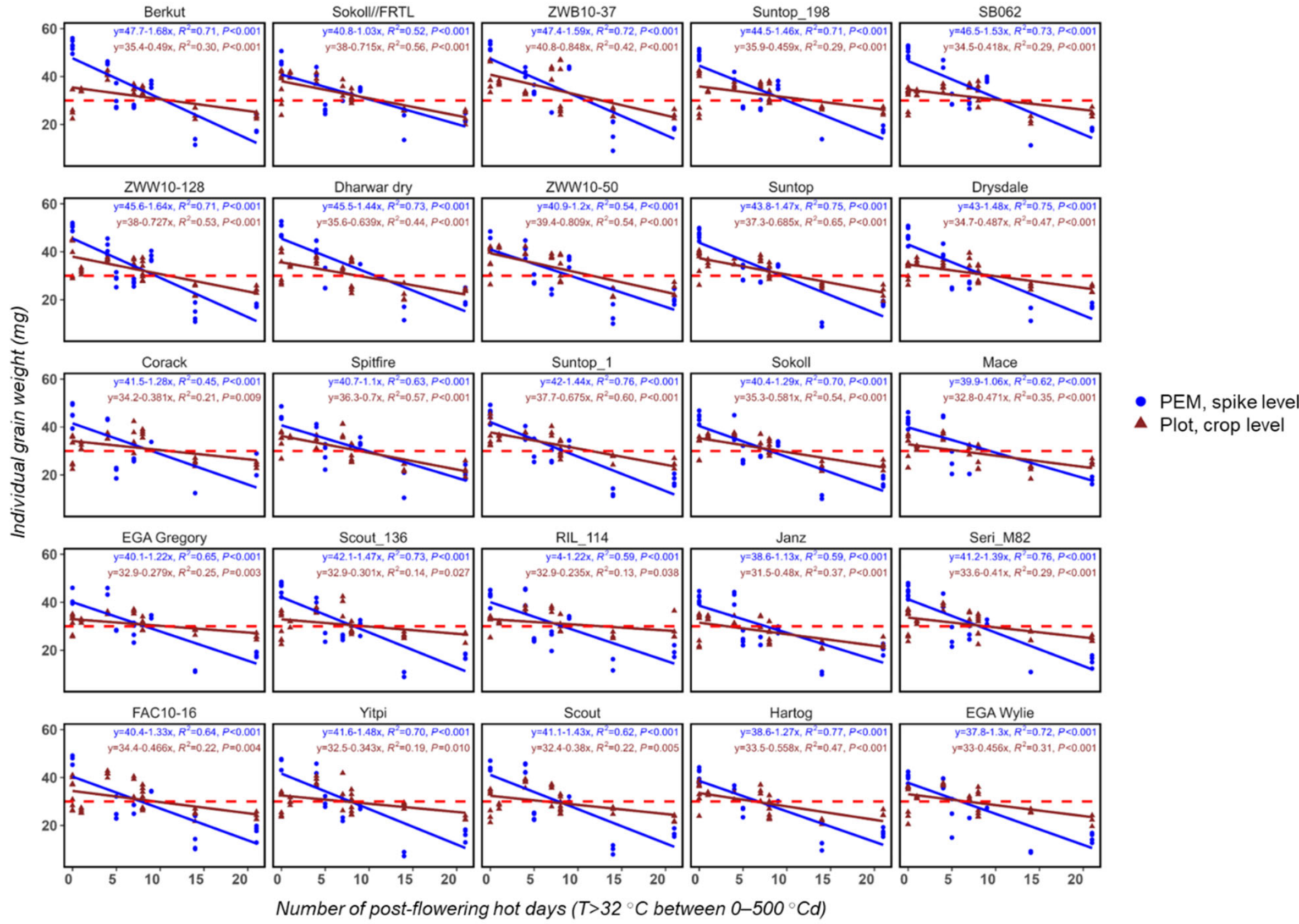

Supplementary Figure S3. Response of individual grain weight to post-flowering hot days for the 25 studied genotypes in PEM trials (spike level) and plot trials (crop level). Reaction norms are expressed relative to the number of hot days (T>32 °C) occurring between 0 and 500 °Cd after flowering. PEM spike-level data is represented by blue circles, and plot data (crop level) by brown triangles. Each point corresponds to raw data of each plot for each genotype in each trial. The red dashed horizontal line corresponds to the 30 mg IGW threshold.

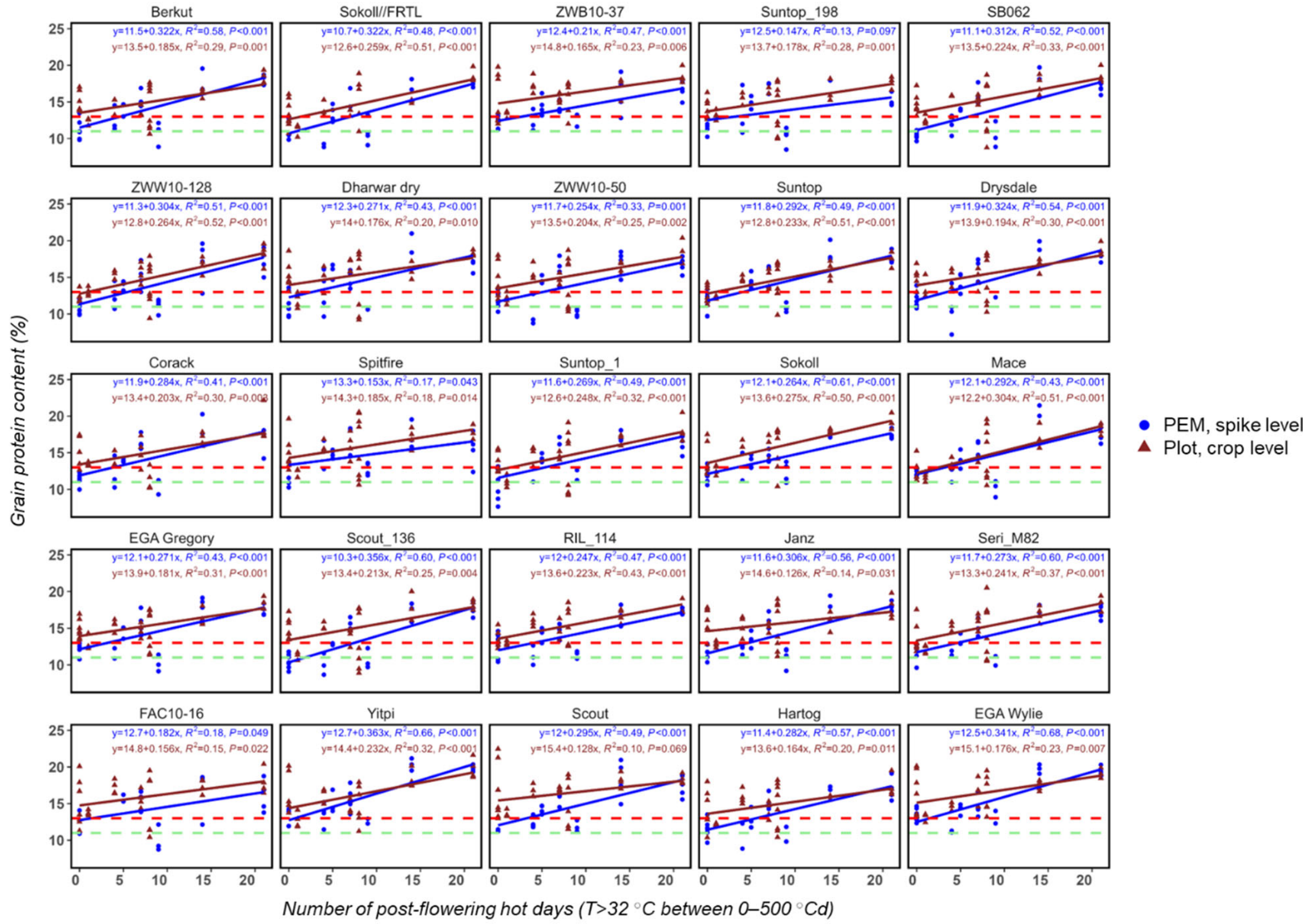

Supplementary Figure S4. Grain protein content (GPC) response to post-flowering hot days for the 25 studied genotypes in PEM trials (spike level) and plot trials (crop level). Reaction norms are expressed relative to the number of hot days (T>32 °C) occurring between 0 and 500 °Cd after flowering. PEM spike-level data is represented by blue circles, and plot data (crop level) by brown triangles. Each point corresponds to raw data of each plot for each genotype in each trial. The green and red dashed horizontal line corresponds to 13 and 15% GPC benchmark, respectively.

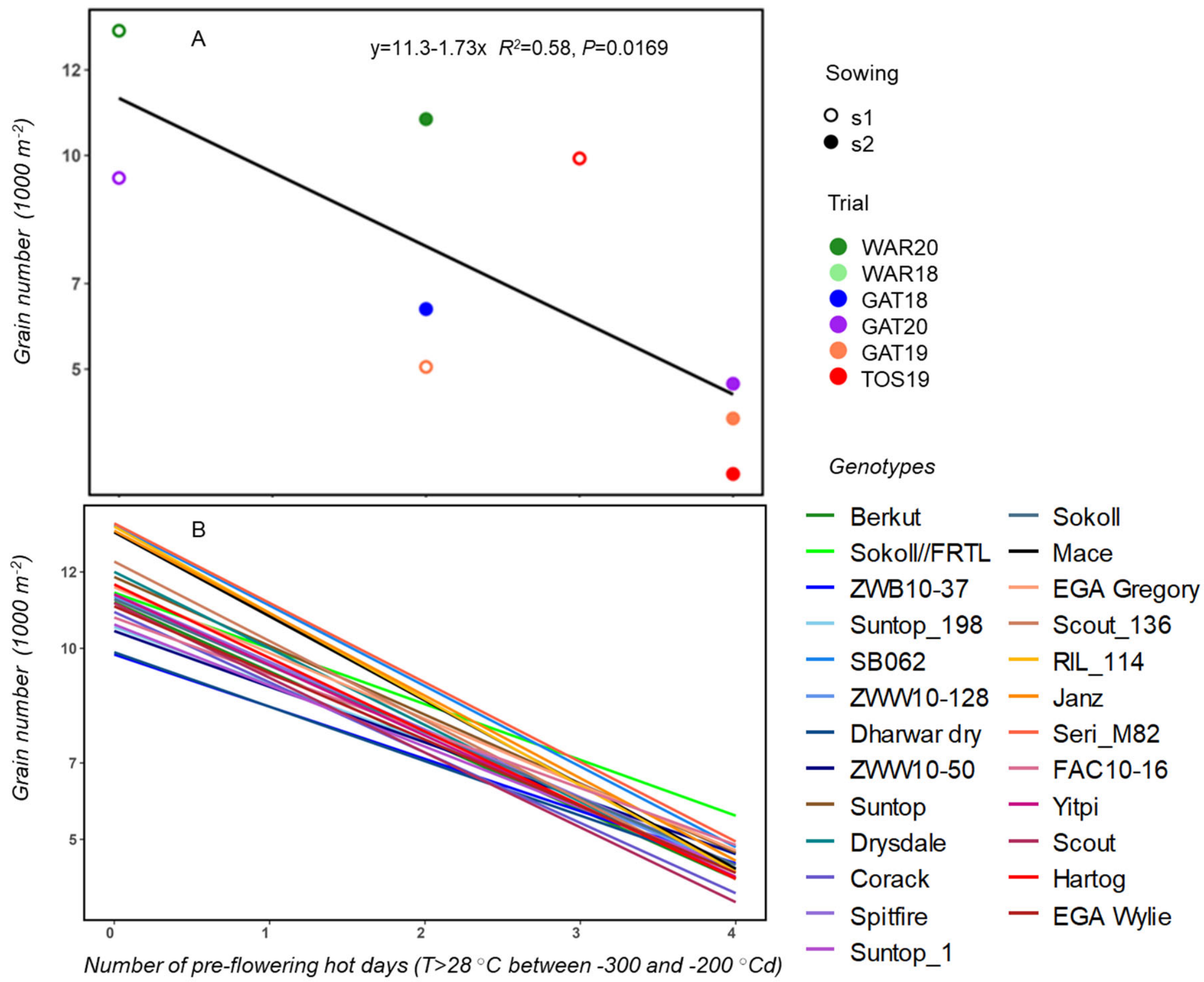

Supplementary Figure S5. Response of grain number to pre-flowering hot days for (A) mean-trial values, and (B) each of the 25 studied genotypes in plot trials. Reaction norms are expressed relative to the number of hot days (T>28 °C) occurring between -300 and -200 °Cd before flowering.

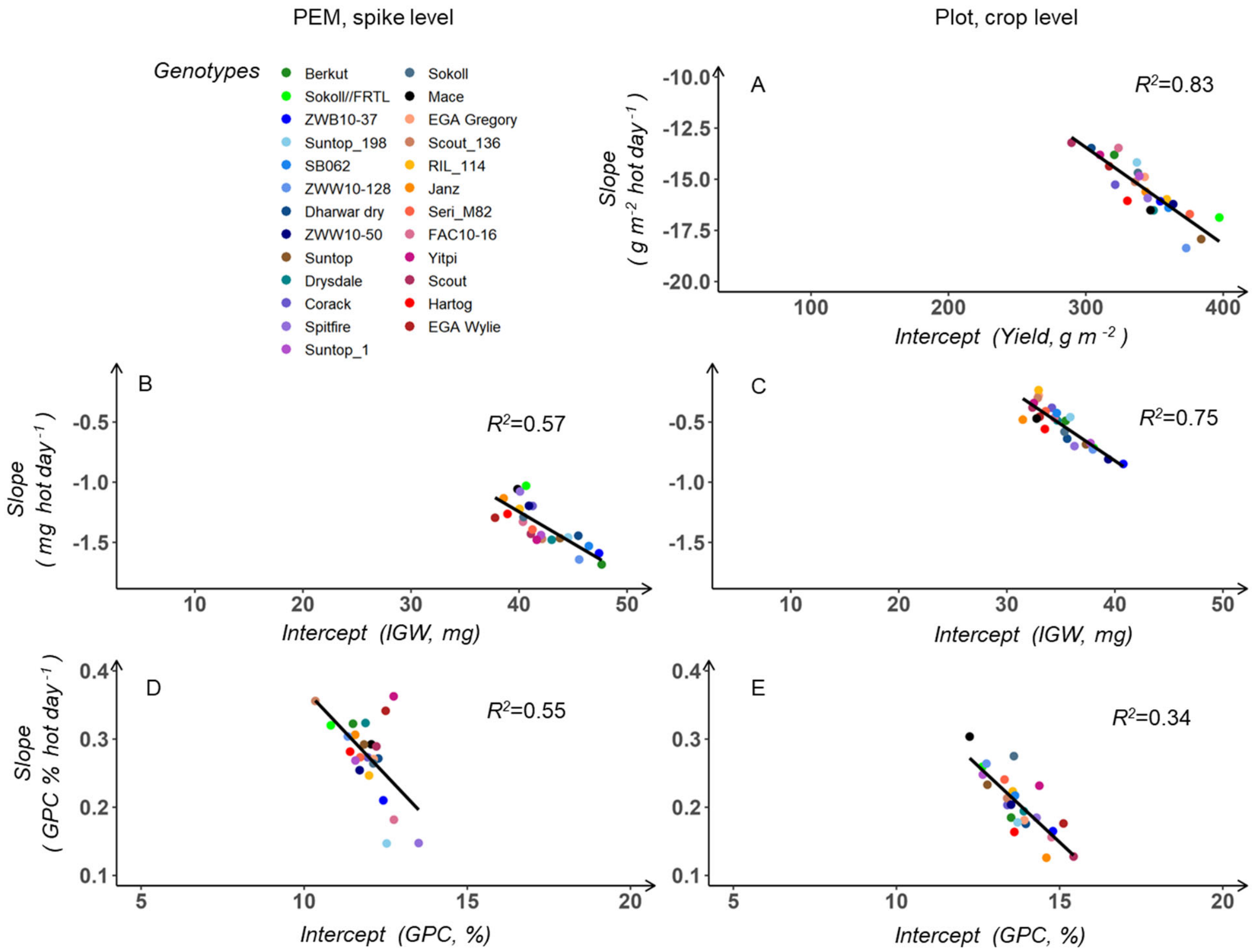

Supplementary Figure S6. Relationships between the slope and intercept values of the reaction norms of (A) yield, (B-C) individual grain weight (IGW) and (D-E) grain protein content (GPC) in response to the number of post-flowering hot days in PEM trials (spike level; B, D) and plot trials (crop level; A, C, E). Slope and intercepts values are presented in **Supplementary Table S4 and Fig. S7**.

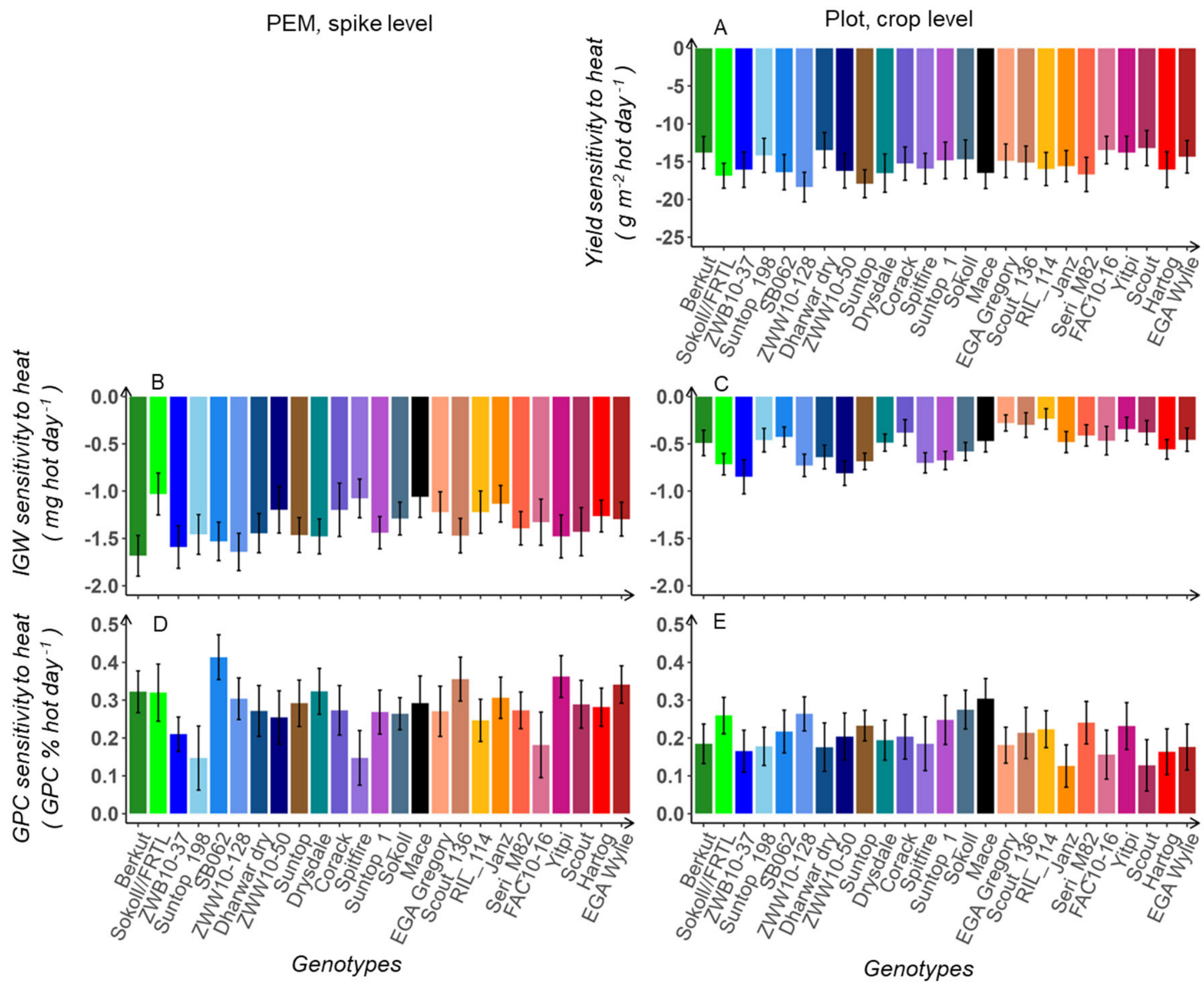

Supplementary Figure S7. Heat sensitivity of (A) yield, (B-C) individual grain weight (IGW) and (D-E) grain protein content (GPC) in PEM trials (spike level; B, D) and plot trials (crop level; A, C, E). Bar graphs represent the estimated slopes of heat sensitivity for each genotype in response to the number of hot days (T>32 °C) occurring between 0 and 500 °Cd after flowering. Error bars indicate standard errors of slope estimates.

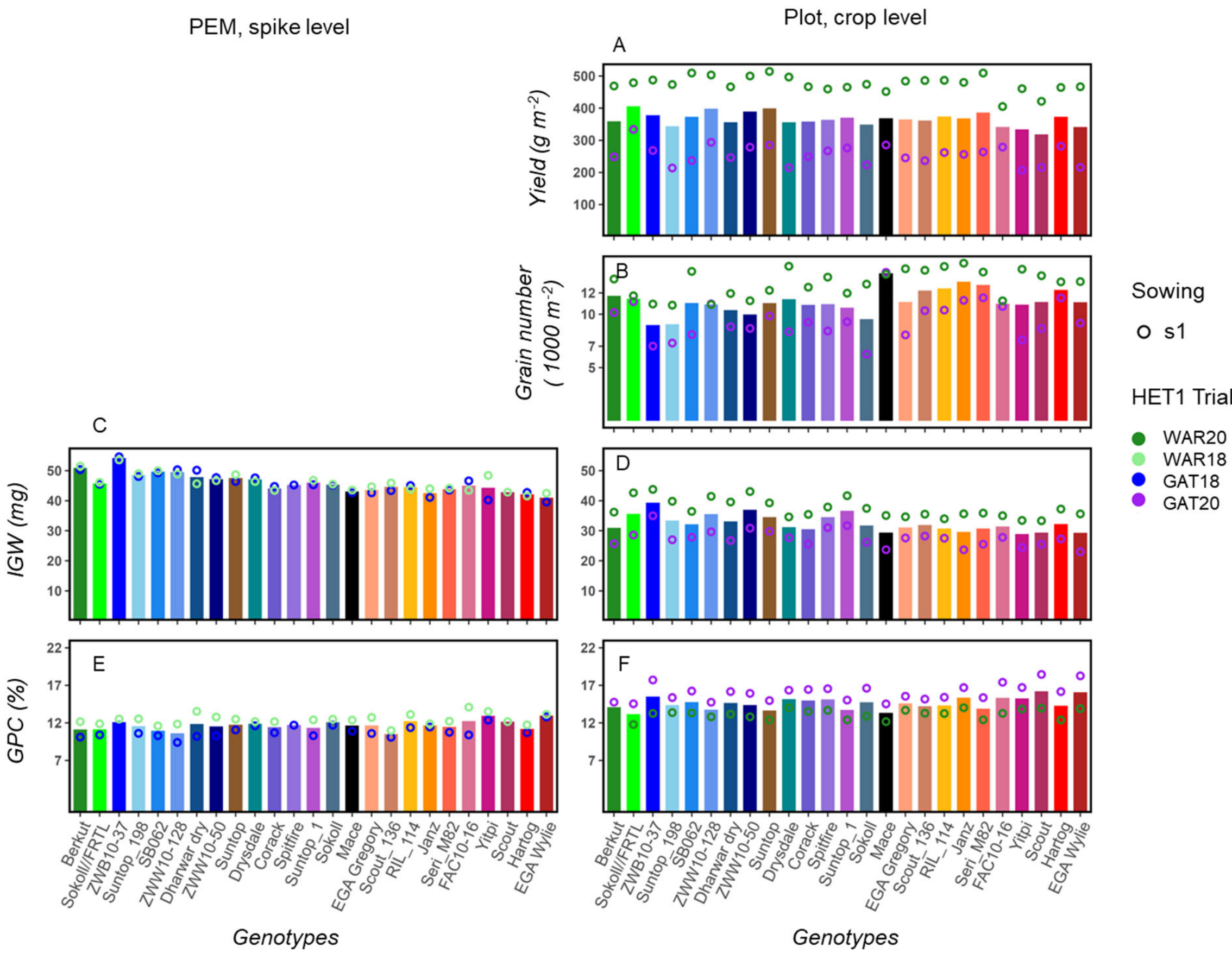

Supplementary Figure S8. Grain yield, grain number, individual grain weight (IGW), and grain protein content (GPC) for the 25 studied genotypes in PEM and plot trials with no or limited heat stress (i.e. HET1). Data points correspond the genotype average within a trial, while bars represent the genotype average across HET1 trials (GAT18s1 and WAR18s1 for PEM trials; GAT20s1 and War20s1 for plot trials).

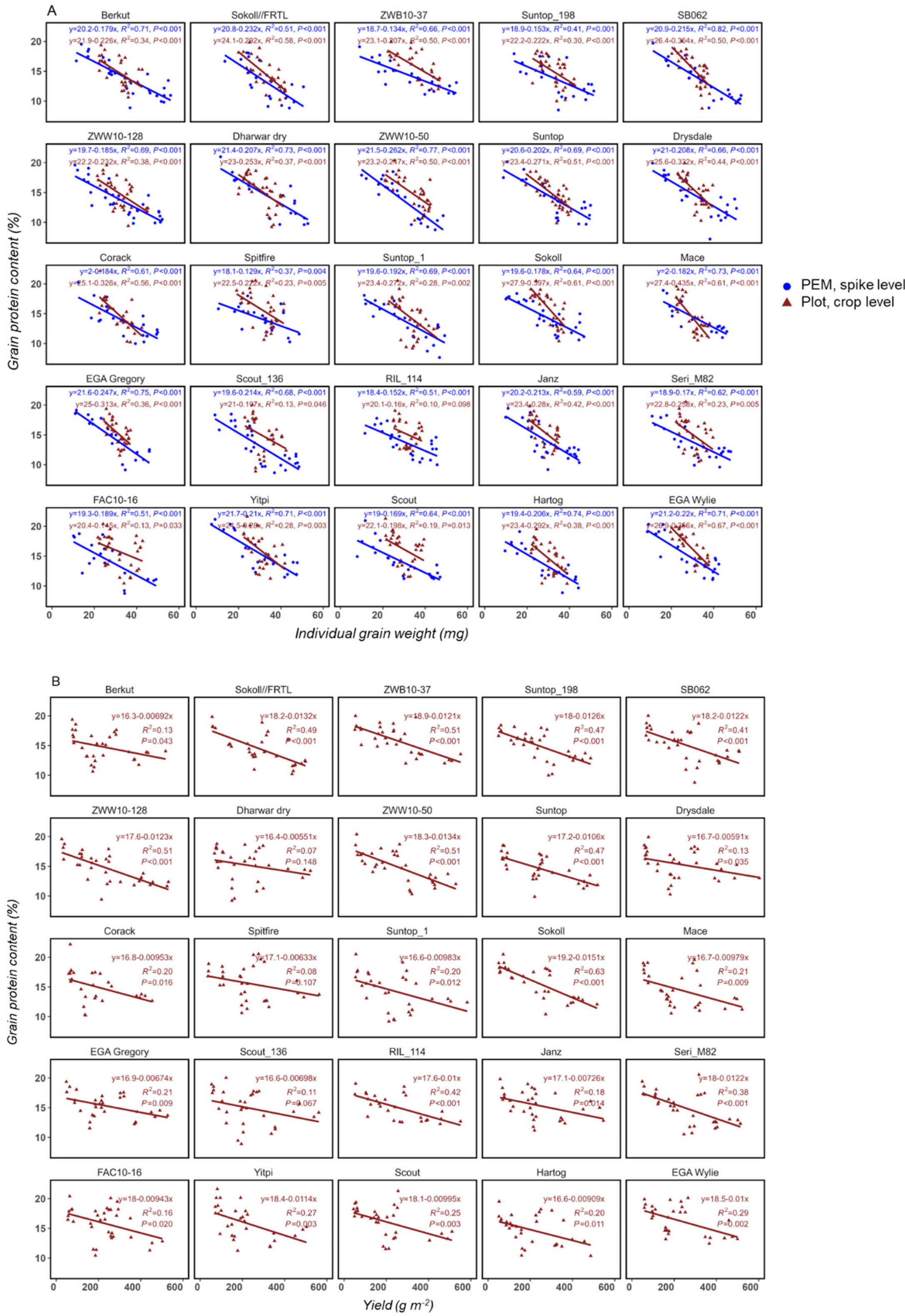

Supplementary Figure S9. Relationship between grain protein content (GPC) and (A) individual grain weight (IGW) in PEM trials (spike level) and plot trials (crop level), and (B) grain yield in conventional plots. Each data point corresponds to raw data of each plot for each genotype in each trial. PEM spike-level data are represented blue circles, and plot crop-level data in brown triangles.

References

Christopher JT, **Manschadi AM**, **Hammer GL**, **Borrell AK**. 2008. Developmental and physiological traits associated with high yield and stay-green phenotype in wheat. *Australian Journal of Agricultural Research* 59, 354–366. https://doi.org/10.1071/AR07193

Christopher M, **Paccapelo V**, **Kelly A**, **Macdonald B**, **Hickey L**, **Richard C**, **Verbyla A**, **Chenu K**, **Borrell A**, **Amin A**, **Christopher J**. 2021. QTL identified for stay-green in a multi-reference nested association mapping population of wheat exhibit context dependent expression and parent-specific alleles. *Field Crops Research* 270, 108181. https://doi.org/10.1016/j.fcr.2021.108181

Condon AG, **Richards RA**, **Rebetzke GJ**, **Farquhar GD**. 2004. Breeding for high water-use efficiency. *Journal of Experimental Botany* 55, 2447–2460. https://doi.org/10.1093/jxb/erh277.

Collins B, **Chapman S**, **Hammer G**, **Chenu K**. 2021. Limiting transpiration rate in high evaporative demand conditions to improve Australian wheat productivity. *in silico Plants* 3, diab006. https://doi.org/10.1093/insilicoplants/diab006

Dinglasan E, **Godwin ID**, **Mortlock MY**, **Hickey LT**. 2016. Resistance to yellow spot in wheat grown under accelerated growth conditions. *Euphytica* 212, 303–315. https://doi.org/10.1007/s10681-016-1660-z

Dreccer MF, **van Herwaarden AF**, **Chapman SC**. 2009. Grain number and grain weight in wheat lines contrasting for stem water soluble carbohydrate concentration. *Field Crops Research* 112, 43–54. https://doi.org/10.1016/j.fcr.2009.02.006

Fletcher A, **Christopher J**, **Hunter M**, **Rebetzke G**, **Chenu K**. 2018. A low-cost method to rapidly and accurately screen for transpiration efficiency in wheat. *Plant Methods* 14, 77. https://doi.org/10.1186/s13007-018-0339-y

GRDC. 2018. 2019 Wheat variety sowing guide for Western Australia, 1–44.

Hickey LT, **Dieters MJ**, **DeLacy IH**, **Kravchuk OY**, **Mares DJ**, **Banks PM**. 2009. Grain dormancy in fixed lines of white-grained wheat (Triticum aestivum L.) grown under controlled environmental conditions. *Euphytica* 168, 303–313. https://doi.org/10.1007/s10681-009-9929-0

Manschadi AM, **Hammer GL**, **Christopher JT**, **DeVoil P**. 2008. Genotypic variation in seedling root architectural traits and implications for drought adaptation in wheat (Triticum aestivum L.). *Plant and Soil* 303, 115–129. https://doi.org/10.1007/s11104-007-9492-1

Olivares-Villegas JJ, **Reynolds MP**, **McDonald GK**. 2007. Drought-adaptive attributes in the Seri/Babax hexaploid wheat population. *Functional Plant Biology* 34, 189–203. https://doi.org/10.1071/FP06148

Rattey A, **Shorter R**, **Chapman S**, **Dreccer MF**, **Van Herwaarden A**. 2009. Variation for and relationships among biomass and grain yield component traits conferring improved yield and grain weight in an elite wheat population grown in variable yield environments. *Crop and Pasture Science* 60, 717–729. https://doi.org/10.1071/CP08460

Rebetzke GJ, **Chapman SC**, **McIntyre CL**, **Richards RA**, **Condon AG**, **Watt M**, **van Herwaarden AF**. 2009. Grain yield improvement in water-limited environments. *Wheat Science and Trade*, 215–249. https://doi.org/10.1002/9780813818832.ch11

Richards RA. 2006. Physiological traits used in the breeding of new cultivars for water-scarce environments. *Agricultural Water Management* 80, 197–211. https://doi.org/10.1016/j.agwat.2005.07.013

Thistlethwaite RJ, **Tan DKY**, **Bokshi AI**, **Ullah S**, **Trethowan RM**. 2020. A phenotyping strategy for evaluating the high-temperature tolerance of wheat. *Field Crops Research* 255, 107905. https://doi.org/10.1016/j.fcr.2020.107905

Ullah N, **Chenu K**. 2019. Impact of post-flowering heat stress on stay-green and grain development in wheat. In: *Proceedings of the 19th Australian Agronomy Conference*, Wagga Wagga, NSW, Australia, 25–29 August 2019.

Zheng Z, **Kilian A**, **Yan G**, **Liu C**. 2014. QTL conferring fusarium crown rot resistance in the elite bread wheat variety EGA Wylie. *PLoS ONE* 9, e96011. https://doi.org/10.1371/journal.pone.0096011
